# Multiplexed activation in mammalian cells using a split-intein CRISPR/Cas12a based synthetic transcription factor

**DOI:** 10.1093/nar/gkab1191

**Published:** 2021-12-15

**Authors:** James W Bryson, Jamie Y Auxillos, Susan J Rosser

**Affiliations:** Department of Quantitative Biology, Biochemistry and Biotechnology, University of Edinburgh, Edinburgh, UK; Centre for Synthetic and Systems Biology and UK Centre for Mammalian Synthetic Biology, School of Biological Sciences, University of Edinburgh, Edinburgh, UK; Institute of Cell Biology, School of Biological Sciences, University of Edinburgh, Edinburgh, UK; Department of Quantitative Biology, Biochemistry and Biotechnology, University of Edinburgh, Edinburgh, UK; Centre for Synthetic and Systems Biology and UK Centre for Mammalian Synthetic Biology, School of Biological Sciences, University of Edinburgh, Edinburgh, UK

## Abstract

The adoption of CRISPR systems for the generation of synthetic transcription factors has greatly simplified the process for upregulating endogenous gene expression, with a plethora of applications in cell biology, bioproduction and cell reprogramming. The recently discovered CRISPR/Cas12a (Cas12a) systems offer extended potential, as Cas12a is capable of processing its own crRNA array, to provide multiple individual crRNAs for subsequent targeting from a single transcript. Here we show the application of dFnCas12a-VPR in mammalian cells, with the *Francisella novicida* Cas12a (FnCas12a) possessing a shorter PAM sequence than *Acidaminococcus* sp. (As) or *Lachnospiraceae bacterium* (Lb) variants, enabling denser targeting of genomic loci, while performing just as well or even better than the other variants. We observe that synergistic activation and multiplexing can be achieved using crRNA arrays but also show that crRNAs expressed towards the 5′ of 6-crRNA arrays show evidence of enhanced activity. This not only represents a more flexible tool for transcriptional modulation but further expands our understanding of the design capabilities and limitations when considering longer crRNA arrays for multiplexed targeting.

## INTRODUCTION

Synthetic transcription factors are modular proteins composed of DNA binding domains and transactivation domains, which enable up-regulation of targeted genes. Whilst a number of strategies have been developed (1), the application of clustered regularly interspersed palindromic repeats (CRISPR) systems has greatly reduced the costs and complexity associated with generating synthetic transcription factors for targeting different loci (2).

A hybridised CRISPR RNA (crRNA) and trans-activating crRNA (tracrRNA) enables targeting of the CRISPR associated protein 9 (Cas9) to a specific locus (3). The spacer sequence within the crRNA confers target specificity, with binding and cleavage only occurring if the spacer sequence is complementary to the target sequence. There must also be a protospacer adjacent motif (PAM) sequence, which varies based on the Cas9 species of origin, adjacent to the target sequence. If the PAM sequence is present, then Cas9 can transiently melt the DNA to enable infiltration by the spacer sequence (4). Subsequently, if the spacer is complementary to the target sequence, then Cas9 will bind and cleave the target DNA.

The generation of a DNase inactive Cas9 variant (dCas9) has subsequently enabled the creation of RNA-guided DNA binding domains, where the specificity of genome targeting can be altered by changing the 20 nt spacer sequence within a single guide RNA (sgRNA) composed of a fused crRNA and tracrRNA (5). A number of groups have subsequently generated synthetic transcription factors by fusing transactivation domains to dCas9 (6,7). Cas9 derived synthetic transcription factors have been employed for a number of applications including; genetic circuits (8), cell reprogramming (9) and biosensors (10).

It is important to note that Cas9 represents only one of a variety of known CRISPR systems, with others possessing divergent and useful properties. In particular Cas12a (formerly Cpf1), similarly to Cas9, functions as an RNA-guided homing endonuclease. However, unlike Cas9, Cas12a can be targeted by a single crRNA (∼40 nucleotides (nt)) as opposed to requiring a combined crRNA and tracrRNA (∼100 nt) (11). Furthermore, in contrast to Cas9, Cas12a possesses RNase activity and can recognise and process an array of adjacent crRNAs within a single transcript to enable targeting of the protein to multiple unique loci (12).

Whilst work has been carried out to generate synthetic transcription factors using DNase dead dCas12a variants from *Acidaminococcus* sp. BV3L6 and *Lachnospiraceae bacterium* ND206 (AsCas12a and LbCas12a respectively) (13), the related *Francisella novicida* variant (FnCas12a) has remained understudied, as initial work suggested it may not cut DNA *in vivo* (11). However, subsequent work by Kim *et al.* showed it did possess activity in mammalian cells (14). FnCas12a was initially characterised as having a shorter PAM sequence than AsCas12a or LbCas12a *in vitro*, ‘TTN’ for the Fn variant compared to ‘TTTN’ for the As and Lb variants. (11). Subsequent cleavage assays in mammalian cells has shown a PAM sequence ‘K(G/T)Y(C/T)TV(A/C/G)’ enables optimal targeting for FnCas12a (15), compared to ‘TTTV’ for both AsCas12a and LbCas12a (16). ‘KYTV’ can on average be found every 21 nt. This targeting density is highly comparable to the targeting capacity of SpCas9 which has a PAM sequence ‘NGG’, which can on average be found every 16 nt. In contrast, the As/LbCas12a PAM sequence ‘TTTV’ can only be found on average every 85 nt.

The ability to target more synthetic transcription factors to a specific genomic region becomes essential in cases where narrow windows of targeting are optimal and in particular, when carrying out multiplexed targeting. One clear example is the case of gene network manipulation, where; (i) there is a 350 nt window within the promoter region where optimal transactivation is observed (17), (ii) multiple promoters will be simultaneously targeted and (iii) multiple studies including this work show that targeting more than one copy of the synthetic transcription factor to the same promoter can enable enhanced transactivation (13,18).

In the following work, we show that FnCas12a can be engineered and applied as a synthetic transcription factor in mammalian cells, by fusing the VPR domain composed of a tri-partite activator containing VP64, P65 and Rta to the C-terminus (19). We subsequently explore whether dFnCas12a-VPR shows orthogonality when screened alongside dAsCas12a-VPR and dLbCas12a-VPR. We then test whether single crRNAs are sufficient for gene activation and look for synergistic transactivation when multiple crRNA target a single promoter. We further explore multiplexed activation from single crRNA arrays as well as assessing the role of position of targeting crRNA within 6-crRNA arrays on the capacity to transactivate targeted genes. Finally, we explore the generation of split intein versions of dFnCas12a-VPR, which provides a strategy for reducing the size of coding sequences that need to be packaged within delivery vectors such as the therapeutically relevant but highly size constrained AAV vector. We test four split intein versions and show that one retains significant activity for all targeted genes.

## MATERIALS AND METHODS

### Plasmid construction

Isothermal mutagenesis was used to introduce amino acid substitutions; D908A for AsCas12a, D917A for FnCas12a and D832A for LbCas12a. Mutation of this highly conserved amino acid has previously been shown to abolish DNase activity (11). The VPR transactivation domain was subcloned onto the 3′ of each dCas12a from dCas9-VPR, restriction ligation.

Single crRNA plasmids and crRNA arrays were generated by first annealing oligos ordered from IDT (Integrated DNA Technologies). The annealed oligos were then ligating into the BpiI (Thermo Scientific cat #ER1012) digested pU6 plasmid backbone using 1 μl of T4 PNK (NEB cat #M0201L) and 1 μl of T4 ligase (NEB cat #M0202L) in a 20 μl reaction, incubated at 37°C for 30 min before transforming into *Escherichia coli*. Sequences for targeting gRNAs and crRNAs are included in Supplementary Table S1.

### Designing split intein constructs

Four split intein locations were chosen based upon the published FnCas12a protein structure (PDB: 5NFV) (20), with locations chosen to be close to the median amino acid and lying within exposed flexible loops within the protein structure. The split inteins NpuN and SspC cloned onto the C-terminus and N-terminus of the first and second halves respectively using Gibson assembly. The coding sequences for each of the split constructs are provided in Supplementary Table S3.

### Cell culturing and transfection

HEK293 cells were cultured in DMEM (Gibco; Life Technologies) with 10% FBS (Gibco; Life Technologies), 4mM glutamine and 1% penicillin-streptomycin (Gibco; Life Technologies). Transfections were carried out a day after seeding ∼200 000 cells per well into 24-well plates. Transfections were performed using either lipofectamine 2000 (luciferase assays) or GenJet In vitro transfection reagent (qRT-PCRs). For the initial luciferase assays 200 ng of the synthetic transcription factor was transfected with 100 ng of the gRNA/crRNA plasmids. For the qRT-PCR assays 500 ng of the synthetic transcription factor was transfected with 250 ng of the gRNA/crRNA plasmids.

### Dual luciferase assay

HEK293 cells were transfected with a Firefly luciferase reporter construct, *Renilla* luciferase normalising construct and the respective synthetic transcription factor construct, with or without a targeting crRNA/gRNA plasmid. Firefly luciferase expression and *Renilla* luciferase expression were then assessed using the dual-luciferase kit (Promega E1910) with measurements carried out with the Modulus II microplate reader (Turner Biosystems). In all cases cells were lysed in passive lysis buffer 48 hours after transfection.

### RNA extraction and cDNA generation

72 hours after transfection cells were harvested and RNA extraction was performed using E.Z.N.A Total RNA Kit 1 (Omega Biotek cat #R6834-01). cDNA generation was performed using SuperScript IV Reverse Transcriptase (Invitrogen). 1 μg of RNA was mixed with 1 μl of 50μM oligo d(T)20 (IDT), 1 μl of 10mM dNTP mix (Promega cat #U1240) and DEPC water up to a final volume of 13 μl in a PCR tube and incubated at 65°C for 5 min then on ice for 1 min. The following components were added to each sample: 4 μl of SuperScript IV Reverse Transcriptase buffer, 1 μl of 0.1 M DTT, 1 ul of dH20, 0.5 μl of RiboLock RNase Inhibitor (Invitrogen) and 0.5 μl of SuperScript IV Reverse Transcriptase (Invitrogen). The reactions were then incubated at 52°C for 10 min followed by 80°C for 10 min and holding at 4°C.

Unless otherwise stated, cells were harvested three days post transfection using E.Z.N.A. Total RNA Kit 1 (Omega Biotek cat #R6834-01) according to the manufacturer's instructions. The concentration and RNA quality was assessed using the nanodrop.

1 μl of 50 μM oligo d(T)20 (IDT) and 1 μl of 10 mM dNTP mix (Promega cat #U1240) were combined with 1 μg of RNA before adding dd H_2_O up to a final volume of 13 μl. cDNA was then generated using SuperScript IV Reverse Transcriptase (Thermo Fisher cat #18090050) according to the manufacturer's instructions using 0.5 μl of RiboLock RNase Inhibitor (Thermo Scientific cat #EO0381) and 0.5 μl of SuperScript IV Reverse Transcriptase per sample.

### qRT-PCR

The 96-well qPCR reaction plates were set up using the Power SYBR Green qPCR mix (Thermo Fisher cat #4367659) according to the manufacturer's protocol, using a 10μl total reaction volume. The 96-well qPCR plate was run on the StepOnePlus real-time PCR machine (Thermo Fisher cat #4376600). Cycle threshold (Ct) values were calculated on the StepOnePlus PCR machine software and further analysed using the statistical analysis software, Prism 8. Primers (against target and normalising genes) utilised in the study are outlined in Supplementary Table S2.

384-well qPCR reaction plates for the multiplexed activation of endogenous genes (Figures 5 and 7) were set up using the Brilliant III ultra-fast SYBR master mix (Agilent cat #600882) according to the manufacturer's protocol, using a 4μl total reaction volume. Samples were loaded on a 384-multiwell plate (Roche cat #04729749001) and ran on the Lightcycler 480 qPCR machine. The Ct values were obtained from the Lightcycler software. ΔCt was calculated by subtracting the mean Ct value for the gene of interest from the mean Ct value for the normalising gene in each experiment. Next the ΔΔCt was calculated by subtracting the ΔCt value for each sample from the ΔCt value for the respective negative control. Finally, relative gene expression was calculated by calculating 2^–ΔΔCt^ for each sample. Subsequent statistical analysis was performed using the statistical analysis software, Prism 8.

## RESULTS

### dFnCas12a-VPR transactivates Luciferase plasmid reporter in mammalian cells

To assess the capability of different Cas12a systems to be adapted as synthetic transcription factors, three variants of Cas12a were chosen. The As, Lb and Fn variants were selected due to their well characterised nature (As and Lb) or the wide targeting range of the PAM sequence (Fn). DNase inactive variants were generated before the VPR transactivation domain was cloned onto the 3′end of each dCas12a.

**Figure 1.**
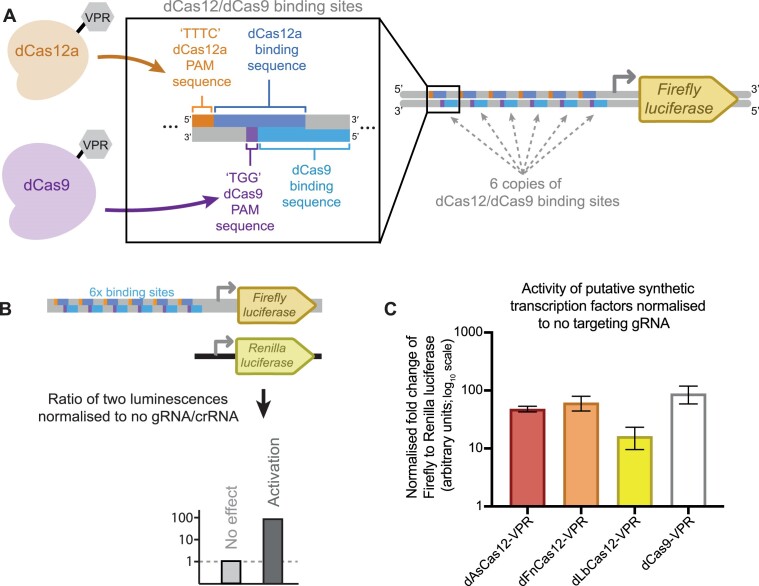
Screening dCas12a-VPR constructs using plasmid-based Firefly luciferase reporter. (**A**) Schematic representation of the targeted promoter region within the Firefly Luciferase reporter plasmid. The top strand of the promoter region contains six repeated binding sequences for the dCas12a constructs (dark blue) with adjacent PAM sequences that can be recognised by all three variants ‘TTTC’ (orange). The bottom strand of the promoter region contains six repeated binding sequences for dCas9-VPR (light blue) with adjacent PAM sequences that can be recognised by dCas9-VPR (purple). (**B**) Diagrammatic representation of the dual luciferase reporter assay. A non-targeted *Renilla* luciferase plasmid was co-transfected with the targeted Firefly luciferase reporter plasmid, to enable normalisation of the relative Firefly luciferase activity between test and control conditions. If a putative synthetic transcription factor was able to transactivate the targeted Firefly luciferase gene, then the ratio of Firefly to *Renilla* luciferase activity would be increased compared to the negative control condition. (**C**) Testing the three dCas12a-VPR variants alongside dCas9-VPR using the dual luciferase assay. Each construct is delivered with a targeting crRNA/gRNA and the resulting ratio is normalised to the ratio when delivered without a crRNA/gRNA. The results represent three biological replicates and the error bars display the SEM.

The three variants (As, Fn and Lb) were initially screened alongside dCas9-VPR using a dual luciferase assay. Utilizing a dual plasmid reporter system (21), each of the dCas12a-VPR constructs and dCas9-VPR were targeted upstream of a Firefly luciferase gene using the respective crRNAs (dCas12a-VPR variants) or sgRNA (dCas9-VPR) (Figure 1A).

The plasmids expressing the synthetic transcription factor and crRNA/sgRNA were co-transfected alongside the targeted Firefly luciferase plasmid and a control *Renilla* luciferase plasmid (Figure 1B) into HEK293 cells. Two days post-transfection the ratio of the targeted Firefly luciferase to the normalising *Renilla* luciferase was measured for each variant with or without a targeting gRNA/crRNA (Supplementary Figure S1) and the ratio for the respective targeted and non-targeted conditions could then be calculated (Figure 1C). Of interest the targeted Fn variant of dCas12a-VPR showed significant transactivation over the non-targeted control (62-fold, *P* = 0.027 based on two-tailed students *t*-test) and displayed non-significantly higher transactivation when compared to the As and Lb variants using a Tukey's multiple comparisons test.

### Orthogonality observed between dCas12a-VPR variants

**Figure 2.**
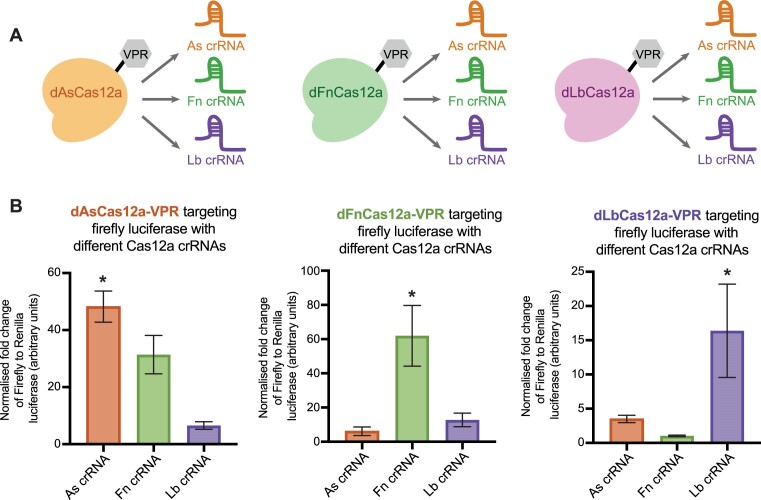
Testing for orthogonality between different dCas12a-VPR variants. (**A**) Schematic representation of the testing of orthogonality using either native (e.g. dAsCas12a-VPR with As crRNA) or non-native (e.g. dAsCas12a-VPR with Fn crRNA) crRNA pairings. (**B**) The three dCas12a-VPR variants (As, Fn and Lb) were screened for orthogonality using the dual luciferase assay (as described in Figure 1B). Each dCas12a-VPR construct was delivered with each of the three targeting crRNA (As, Fn or Lb) or no targeting crRNA. Results display the mean luciferase activity when each of the three targeting crRNAs are transfected, normalised to the mean luciferase for the respective no targeting crRNA condition. Results correspond to three biological replicates, error bars show SEM and the stars (*) denote significant (*P* < 0.05) expression relative to no crRNA based on a Dunnett's multiple comparisons test.

We subsequently sought to test for orthogonality between the three dCas12a-VPR variants (Figure 2A). The activity of each dCas12a-VPR variant when delivered with crRNAs from each of the variants was normalised to transfection without a targeting crRNA, measured using the dual luciferase assay previously described (Figure 1B).

When a Dunnett's multiple comparisons test was performed to test the fold transactivation of the dCas12a-VPR constructs delivered with each targeting crRNA compared to without a crRNA, we see that only the cognate crRNAs show significant transactivation for each of the three variants. Of note dAsCas12a-VPR delivered with the Fn crRNA shows potential albeit non-significant cross reactivity (*P* = 0.17). In contrast dFnCas12a-VPR delivered with the Lb crRNA and dLbCas12a-VPR delivered with the Fn crRNA show particularly low significance (*P* = 0.993 and *P* = 0.999 respectively), suggesting they could serve as an orthogonal pair (Figure 2B).

### Single crRNAs are sufficient for transactivation of endogenous genes

**Figure 3.**
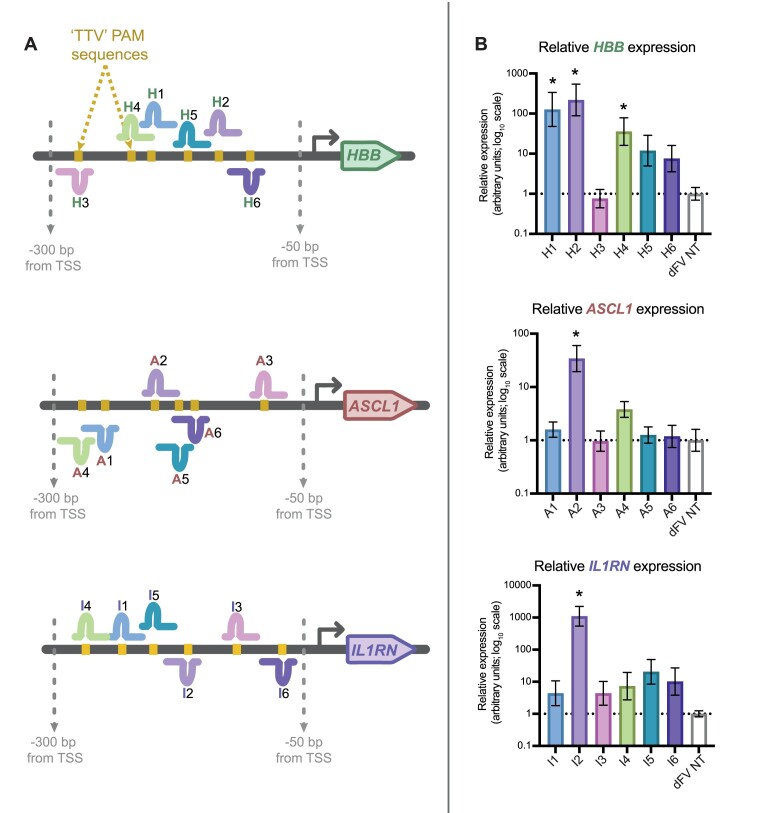
Testing single crRNAs for endogenous gene activation. (**A**) dFnCas12a-VPR was screened for activity targeting three endogenous genes; *HBB*, *ASCL1* and *IL1RN* in HEK293 cells using single crRNAs. The promoter of each gene was targeted with six single crRNAs and transcriptional upregulation was compared to a non-targeting (NT) crRNA. (**B**) The change in transcript abundance was then measured using qRT-PCR, with results shown for the three biological replicates, with the error bars showing SEM. Stars (*) show results with a *P* value < 0.05 after one-way ANOVA followed by a post-hoc Dunnetts test to compare the expression of each targeting crRNA relative to the non-targeting negative control.

Having demonstrated the activity of dFnCas12a-VPR using a plasmid-based reporter, we next sought to test whether transactivation of endogenous genes could be achieved. The three genes *HBB*, *ASCL1* and *IL1RN* were chosen for targeting as they had been found to be especially amenable to transactivation when targeted with dCas9 based synthetic transcription factors (22). Six crRNAs were designed to target each of the three respective promoters. The crRNAs were designed to utilise a ‘TTV’ PAM sequence within a window 50–300 nt upstream of the transcription start site (TSS) (Figure 3A). These TSS were identified using Fantom5 software (23), available within UCSC genome browser, which maps TSSs using capped analysis of gene expression (CAGE) data. This window was selected as previous work had shown maximal transactivation of endogenous genes was obtained when targeting this window with a Cas9 derived synthetic transcription factor (17).

The crRNA plasmids were individually transfected alongside dFnCas12a-VPR into HEK293 cells. Three days post transfection, the total RNA were extracted for qRT-PCR. When assessing the gene expression across all three genes using a Dunnett's multiple comparisons test we found three functional crRNA for *HBB* that showed significant activation (H1, H2 and H4; with *P* = 0.0028, *P* = 0.0011 and *P* = 0.0253 respectively), and one crRNA each for *ASCL1* (A2, with *P* = 0.0003) and *IL1RN* (I2, with *P* = 0.0002) (Figure 3B). These results showed that a single crRNA was sufficient to enable transactivation of endogenous genes.

### Targeting multiple crRNAs enhances transactivation with evidence for synergy

Having shown that targeting dFnCas12a-VPR using single crRNAs was sufficient for transactivation, we next explored if the targeting of multiple crRNAs to the same promoter further enhanced gene expression synergistically. For each gene, the two individual crRNAs that showed the highest fold up-regulation were screened for transactivation, comparing their activity when co-transfected compared to individually transfected. For *HBB* and *IL1RN* a further crRNA pair was selected from crRNAs which had shown either weak or no significant transactivation (H4 + H5 and I4 + I6). To enable assessment of the relative impact of delivering two crRNAs compared to individual crRNAs, equimolar concentrations of crRNA plasmids were delivered to HEK293 cells.

**Figure 4.**
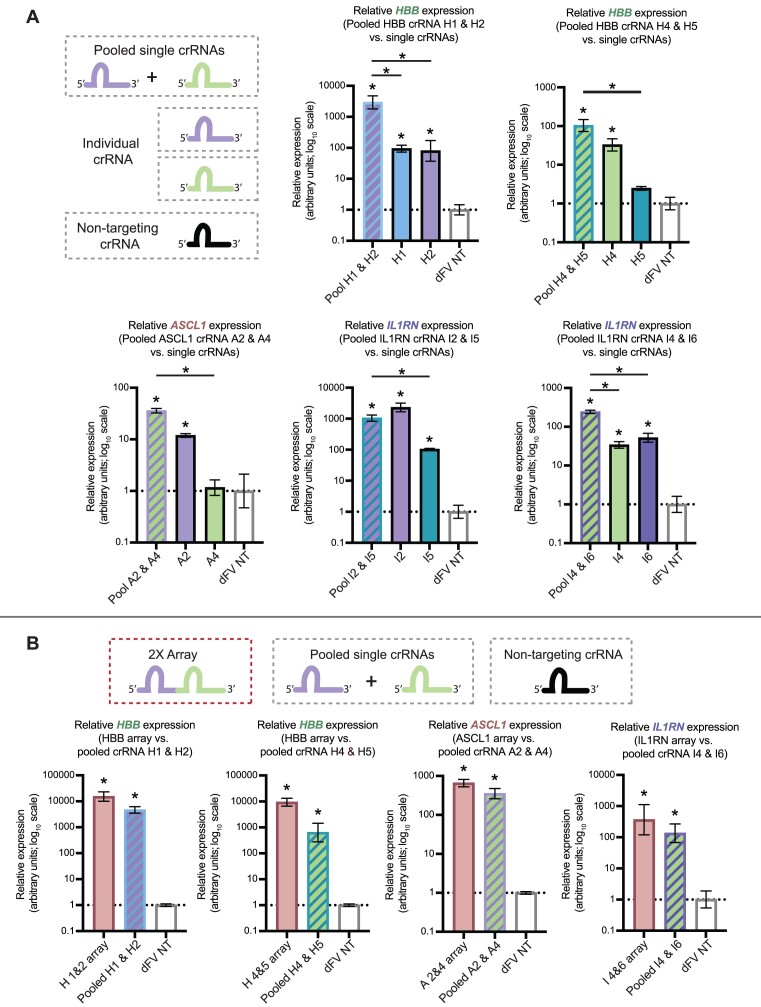
Enhanced activation observed with co-expression of targeting crRNAs. (**A**) The most active individual crRNAs (from Figure 3) were delivered individually or co-transfected into HEK293 cells. The RNA abundance for each targeted gene was then measured by qRT-PCR and normalised to RNA expression when dFnCas12a-VPR was delivered with a non-targeting (NT) crRNA. The pair *IL1RN* crRNA 2 and *IL1RN* crRNA 5 were excluded as the spacer sequences overlapped. Results from three biological replicates were measured by qRT-PCR and normalised to delivery with a non-targeting crRNA, with stars (*) showing results with a *P* value <0.05 based on Tukey's multiple comparison test and error bars showing SEM. (**B**) The crRNA pairs were incorporated into a single 2-crRNA array and the activity of this array was screened compared to the co-transfection of the single crRNAs. Results from three biological replicates were measured by qRT-PCR and normalised to delivery with a non-targeting crRNA, with stars (*) showing results with a *P* value <0.05 based on Dunnett's multiple comparison test and error bars showing SEM.

Analysis by qRT-PCR showed the mean increase in mRNA abundance for the co-transfected condition was consistently higher than the most active individual crRNA (Figure 4A), with one notable exception (*IL1RN* crRNA 2 + 5) discussed below. When a two tailed *t*-test was performed between the co-transfected and most active individual crRNA conditions, we saw a significant increase in mRNA abundance for; *HBB* crRNA 1 + 2 (*P* = 0.017), *ASCL1* crRNA 2 + 4 (*P* = 0.001), and *IL1RN* crRNA 4 + 6 (*P* = 0.005). We also observed a non-significant increase in mRNA abundance for *HBB* crRNA 4 + 5 (*P* = 0.0875). For one of the tested crRNA pairs (*IL1RN* crRNA 2 and 5) the spacer sequences had partial complementarity (12 nucleotides). This may explain the small decrease in mRNA abundance observed when comparing co-transfection to *IL1RN* crRNA 2 individually (non-significant, *P* = 0.117). As a result, the *IL1RN* crRNA 2 + 5 pair was excluded from subsequent analysis.

As one of the advantages of Cas12a is the capacity to process crRNA arrays, we sought to test whether 2-crRNA arrays, consisting of a pair of crRNAs in tandem, could be utilised by dFnCas12a-VPR for transactivating target genes and whether these short arrays would enable increased or synergistic activation compared to the delivery of individual crRNAs. To test this, 2-crRNA arrays were constructed using the most active crRNA pairs from the preceding experiments. Three days after transfection into HEK293 cells, the fold upregulation induced using these arrays was tested and compared to the co-transfected crRNAs and a non-targeting crRNA control. We consistently observed that the crRNA arrays performed as well if not better than the co-transfected crRNAs, with a higher mean fold upregulation for the arrays compared with the co-transfected crRNAs in all cases (Figure 4B).

We subsequently sought to test the crRNA arrays for evidence of synergistic transactivation. Synergy is here defined as showing greater transactivation than would be expected from adding the transactivation caused by each individual crRNA. To test this a hypothetical additive distribution was calculated by summing the distributions for the single crRNA conditions (Supplementary Note 1). Two tailed t-tests were then used to check whether significantly greater activation was seen for the array conditions than their respective hypothetical additive distributions. We saw that synergy was indeed observed for two of the cases, *HBB* (1 + 2) array (*P* = 0.0027) and *HBB* (4 + 5) array (*P* = 0.0027) (Supplementary Figure S2). Significance was not achieved for the *ASCL1* array (*P* = 0.1891) or the IL1RN array (*P* = 0.1069). These results showed that pairs of targeting crRNA expressed from a single array can induce significant up-regulation, which in some cases was significantly higher than expression observed for the individually delivered crRNAs.

### Multiplexed activation from crRNA arrays

**Figure 5.**
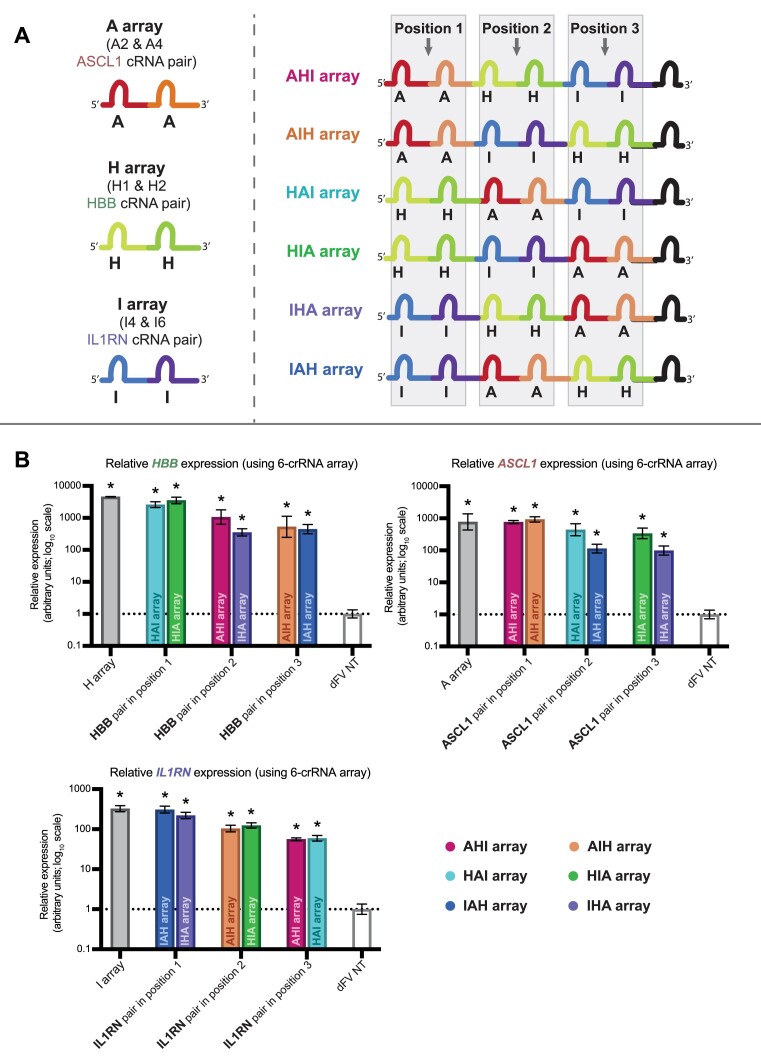
Multiplexed activation of endogenous genes. (**A**) The left panel shows a schematic of the most active crRNA array for targeting each of the three genes from Figure 4B. The right panel shows the designs of the combinatorial 6-crRNA arrays for screening for multiplexed activation. The six arrays were designed to ensure each of the pairs of crRNAs would be present in the first and second, third and fourth or fifth and sixth positions within the 6-crRNA arrays. (**B**) The six arrays were separately transfected into HEK293 cells alongside dFnCas12a-VPR and the expression for each of the three targeted genes was assessed by qRT-PCR, normalising to the expression with a non-targeting crRNA. The grey bars showing the relative expression for the respective targeting 2-crRNA array, serving as a positive control. The results from the three biological replicates are displayed based upon the position of the respective targeting crRNAs within the 6-crRNA arrays, with stars (*) showing results with a *P* value <0.05 based on Dunnett's multiple comparisons test and the error bars displaying SEM.

Having observed that transactivation of individual genes could be achieved using arrays with dFnCas12a-VPR, we next sought to test longer arrays designed to target multiple genes simultaneously, while exploring the impact of crRNA order within the array on activity. To achieve this, the most active crRNA arrays from the previous experiment (Figure 4B) were utilised for the generation of 6-crRNA arrays. Six different arrays were designed with three pairs of crRNAs, such that each pair targeted one of three promoters (Figure 5A). Using this design, all six combinations could be explored to not only test for significant multiplexed activation but also test whether changing the position or flanking crRNA sequences impacted the capacity of a crRNA array to induce transactivation. The constructs were transfected, with the respective targeting 2-crRNA array (grey bars) serving as a positive control. A Dunnett's multiple comparisons test was performed to test whether each 6-crRNA array showed significant transactivation above the negative control. The results showed that each crRNA array was able to induce multiplexed activation, with significantly up-regulated expression for every targeted gene (Figure 5B).

### Modest order dependent array activity observed for individual and paired crRNA

For all three genes targeted, there appeared to be a clear correlation between the position of the targeting crRNAs within the arrays and the fold up-regulation. More specifically when the gene targeting crRNAs were positioned closer to the 3′ end of the array, increases in mRNA abundance were consistently diminished across all three genes tested (Figure 5B). When simple linear regression was performed, a strong inverse correlation between the targeting crRNA position within an array and gene activation of the targeted gene was observed for *IL1RN* (*R*^2^ = 0.8457, *P* < 0.0001), whilst weaker inverse correlations were observed for ASCL1 and HBB (*R*^2^ = 0.4160, *P* = 0.0039 and *R*^2^ = 0.4876, *P* = 0.0013, respectively) (Supplementary Figure S3).

To inspect the relationship of crRNA location and activation strength at a higher resolution, a series of crRNA arrays were generated where each array possessed a single targeting crRNA and five non-targeting crRNAs. The non-targeting crRNAs were rationally designed from different randomly generated 20 nucleotide sequences, that showed no perfect matches against the human genome.

**Figure 6.**
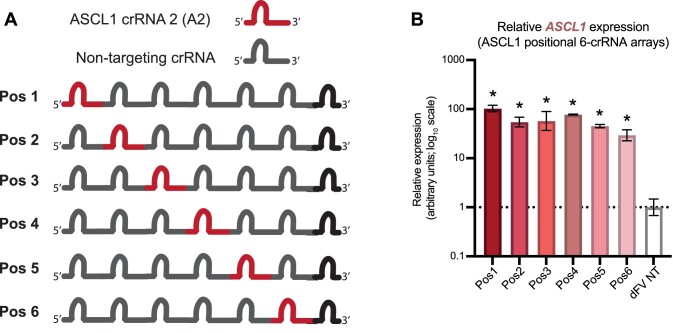
Position dependent activity within pol3 derived crRNA arrays. (**A**) Design of arrays constructed for testing the impact of position for a single targeting crRNA within a 6-crRNA array. (**B**) qRT-PCR analysis of *ASCL1* mRNA abundance for the six arrays after transfection into HEK293 cells, with the graph showing results from the three biological replicates, error bars displaying SEM and stars (*) showing results with a *P* value <0.05 based on a Dunnett's multiple comparison test.

Six different versions of the array were generated so that all positions of the targeting crRNA (*ASCL1* crRNA 2) within the array could be tested (Figure 6A). An initial Dunnett's multiple comparisons test was performed to test whether each of the 6-crRNA arrays showed significant transactivation of *ASCL1* above the negative control. The results showed significant transactivation for all six arrays, however we also observed a reduction in transactivation of *ASCL1* as the targeting crRNA was positioned closer to the 3′ of the array, with the highest mean fold-upregulation (103-fold) when the crRNA was at the first (most 5′) position and the lowest fold up-regulation (29-fold) when the crRNA was at the last position (most 3′) (Figure 6B). When simple linear regression was performed, a weak reduction in activity when the targeting crRNAs were positioned towards the 3′ of the arrays was observed (*R*^2^ = 0.3671, *P* = 0.0077) (Supplementary Figure S4). While a broader trend of reduction in activity is still observed when a single targeting crRNA is expressed at different positions within an array, we see that there are local deviations at some positions (such as Pos4). This may be due to other factors which contribute to crRNA array stability and processing through changes in RNA sequence and structure.

### Split intein dFnCas12a-VPR retains activity for multiplexed transactivation

Having established dFnCas12a-VPR as a potent activator and characterised it in detail, we next sought to enhance its value in biotechnological applications. In its current form the dFnCas12a-VPR coding sequence is too long to be packaged into Adeno-associated viruses, a low immunogenicity delivery vector, for clinical focussed applications (24). To address this, we sought to utilise a chimeric split intein system using the N-terminal fragment of Npu and the C-terminal fragment of SspC (NpuN-SspC) (25). The NpuN-SspC split intein system should enable the dFnCas12a-VPR protein to be expressed as halves from two different constructs before trans-splicing to form a contiguous protein *in vivo* (Supplementary Figure S5A).

**Figure 7.**
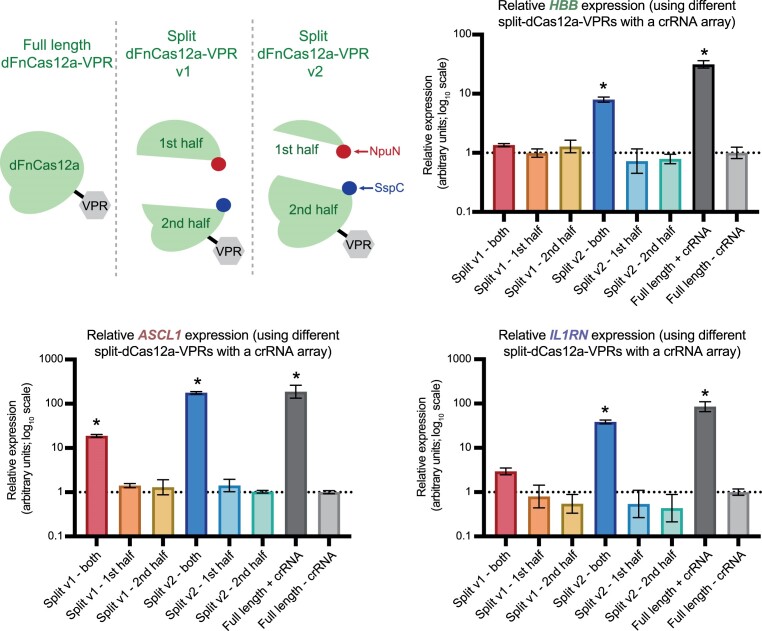
Testing split intein dFnCas12a-VPR for multiplexed transactivation. The two most active split versions from initial testing were screened for activity by transiently transfecting each half or both halves alongside the targeting crRNA array IAH. The graphs display qRT-PCR results from three biological replicates looking at transactivation of the respective genes normalised to the full length dFnCas12a-VPR delivered without a targeting crRNA. The error bars show the SEM and stars show results with a *P* value <0.05 based on a Dunnett's multiple comparison test.

To test this strategy, we generated four rationally designed split versions (further described in methods) (Supplementary Figure S5B), with initial tests suggesting that two of these permitted transactivation (version 1 and version 2 – Supplementary Figure S6). We sought to further test these two versions and see whether they could be used for multiplexed up-regulation from a single crRNA array. To test this, both halves were delivered alongside the IAH array used in the previous experiment (Figure 5B). This was chosen as it enabled comparable fold-changes in activation for all three of the genes targeted. A Dunnett's multiple comparisons test was performed to test whether any of the individual halves or co-delivered halves showed significant transactivation above the negative control (full length dFnCas12a-VPR without a targeting crRNA). The results showed that significant transactivation of all three genes could be achieved with version 2, with version 1 only inducing significant transactivation for two of the targeted genes (Figure 7). As such we show the successful reconstitution of split intein dFnCas12a-VPR for multiplexed transactivation of human genes.

## DISCUSSION

Here, we have shown the first application of engineering FnCas12a derived synthetic transcription factors in mammalian cells. The key advantage of the Fn variant is the simpler PAM sequence ‘KYTV’ when compared to the commonly utilised As or Lb variants ‘TTTV’.

This translates to being able to target on average every 21 nt as opposed to on average every 85 nt, highly comparable to the Cas9 PAM sequence ‘NGG’ which enables targeting on average every 16 nt. This allows much denser targeting, with more potential targets within any given length of DNA. This is of particular interest for transactivation of target genes as we have also highlighted that delivery of multiple active crRNAs targeting the same promoter region further enhances up-regulation, showing evidence of synergistic transactivation for some targets. When screening the three variants for orthogonality, we see evidence for orthogonality in particular for the pairing, dFnCas12a-VPR and dLbCas12a-VPR with no significant transactivation observed when they are co-transfected with the reciprocal crRNA. Such orthogonality is very important when considering applications where two different functionalities are being encoded on two separate proteins. In these cases it is critical that the crRNAs for targeting one of the proteins does not lead to inadvertent targeting of the other protein. For example when looking to induce transactivation of a set of target genes and repression of a different set, cross-talk between the two dCas12a-VPR/crRNA pairs would confound the intended activity.

We found that dFnCas12a-VPR can be used for multiplexed transactivation of three different genes from a single transcript. Due to the relative ease of construction of these crRNA arrays, compared to assembling multiple gRNAs for Cas9 based systems, this simplifies the targeting of multiple genes for simultaneous transactivation. We have consistently observed a reduction in activity for crRNAs positioned closer to the 3′ of crRNA arrays expressed from the U6 pol 3 promoter. This information can inform design constraints when targeting multiple genes for upregulation from a single array. In particular, arrays can be designed to express crRNAs closer to the 5′ end of an array where they target genes that are challenging to upregulate or where higher overexpression is desired. Conversely, crRNAs targeting genes that are easier to up-regulate or where lower over-expression is desired can be positioned closer to the 3′ end of an array.

One likely explanation for the reduction of activity as the crRNA is positioned closer to the 3′ end of the array is that crRNA abundance is reduced. Zetsche *et al.* showed with RNA-seq data a decreased crRNA abundance towards the 3′ of a 3-crRNA array when expressed in HEK293 cells (26). This may be due to the presence of a weak non-canonical Pol III terminator sequence ‘TTTCT’ (27) within all of the direct repeats within the crRNA array.

Whilst previous work has explored the role of PAM selection (28) and spacer sequence choice (29) on crRNA activity, to our knowledge this is the first time where order-dependent activity of hU6 expressed crRNA arrays have been shown. This is of key relevance to researchers as U6 promoters naturally highly express short non-coding RNA, with a defined termination sequence of five thymidines. As such the majority of gRNA/crRNA mammalian expression plasmids utilise this promoter and the findings of order dependent activity will have relevance to researchers working with Cas12a or derived synthetic transcription factors. In particular the order dependent activity of crRNAs within an array also points to an alternate approach for diversifying the fold change of transactivation of different genes within genetic networks and pathways. This may open up opportunities for streamlined manipulation of pathways. For example, the promoters of multiple genes within a pathway can be targeted by the same crRNA but in different orderings. Subsequent sequencing of high production strains can reveal the enrichment of order for specific crRNAs, which in turn reveals when higher transactivation or reduced transactivation of a specific gene within a pathway are being selected for. This in turn can help to reveal key bottlenecks or toxicities that emerge. A similar approach can be considered for processes such as cell reprogramming and indeed any process where transcriptional modulation of a gene network can be correlated with a phenotype.

Finally, we identified a split version of dFnCas12a-VPR, allowing the coding sequence to be expressed separately and reconstituted through post-translational splicing when co-delivered. This not only increases potential avenues for coordinating activity due to the dependence of both halves being expressed, but further by splitting the coding sequence, the sizes of the genetic constructs fall within the packaging constraints of AAV vectors opening future avenues for therapeutic delivery.

Through this expansion of the Cas12a toolkit researchers can simultaneously transactivate multiple genes with ease, with the capacity to densely target multiple promoters. In addition, the compact nature of the crRNA array both facilitates cheap and easy construction using short oligo-based assembly strategies. Furthermore the compact arrays expand the potential of CRISPR systems when considering AAV viral delivery for therapeutic applications especially when paired with our split Cas12a strategy or alternate approaches (30).

## DATA AVAILABILITY

Supplementary figures and information are available at NAR online. The following plasmids will be available on Addgene; dFnCas12a-VPR (#179520), Split v2-NpuN half 1 (#179521) and SspC half2-split v2 (#179522).

## Supplementary Material

gkab1191_Supplemental_File

## Notes

Present address: James W. Bryson, Biotech Research and Innovation Centre, University of Copenhagen, Ole Maaløes Vej 5, Copenhagen N 2200, Denmark.

Present address: Jamie Y. Auxillos, Copenhagen Biocenter, Department of Biology, University of Copenhagen, Ole Maaløes Vej 5, Copenhagen N 2200, Denmark.
