# Supplementary material for: Multiplexed activation in mammalian cells using a split-intein CRISPR/Cas12a based synthetic transcription factor
Source: Nucleic Acids Res. 2021 Dec 15;50(1):549–60. doi: 10.1093/nar/gkab1191 (PMC8754635; doi:10.1093/nar/gkab1191)
Supplement: gkab1191_Supplemental_File [file gkab1191_supplemental_file.pdf]

SUPPLEMENTARY FIGURES

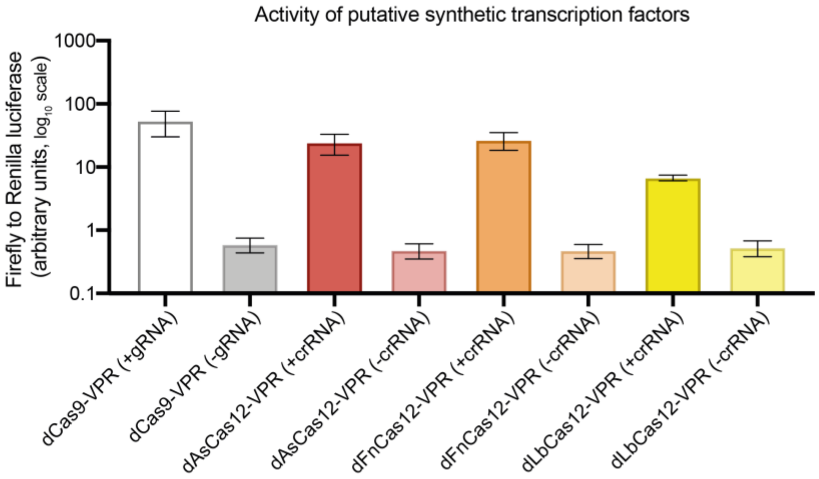

Supplementary Figure 1 - Screening dCas12a-VPR constructs using plasmid-based Firefly luciferase reporter (un-normalised results)

Testing the three dCas12a-VPR variants alongside dCas9-VPR using the dual luciferase assay. Each construct is delivered with or without a targeting crRNA/gRNA. The results represent three biological replicates and the error bars display the SEM.

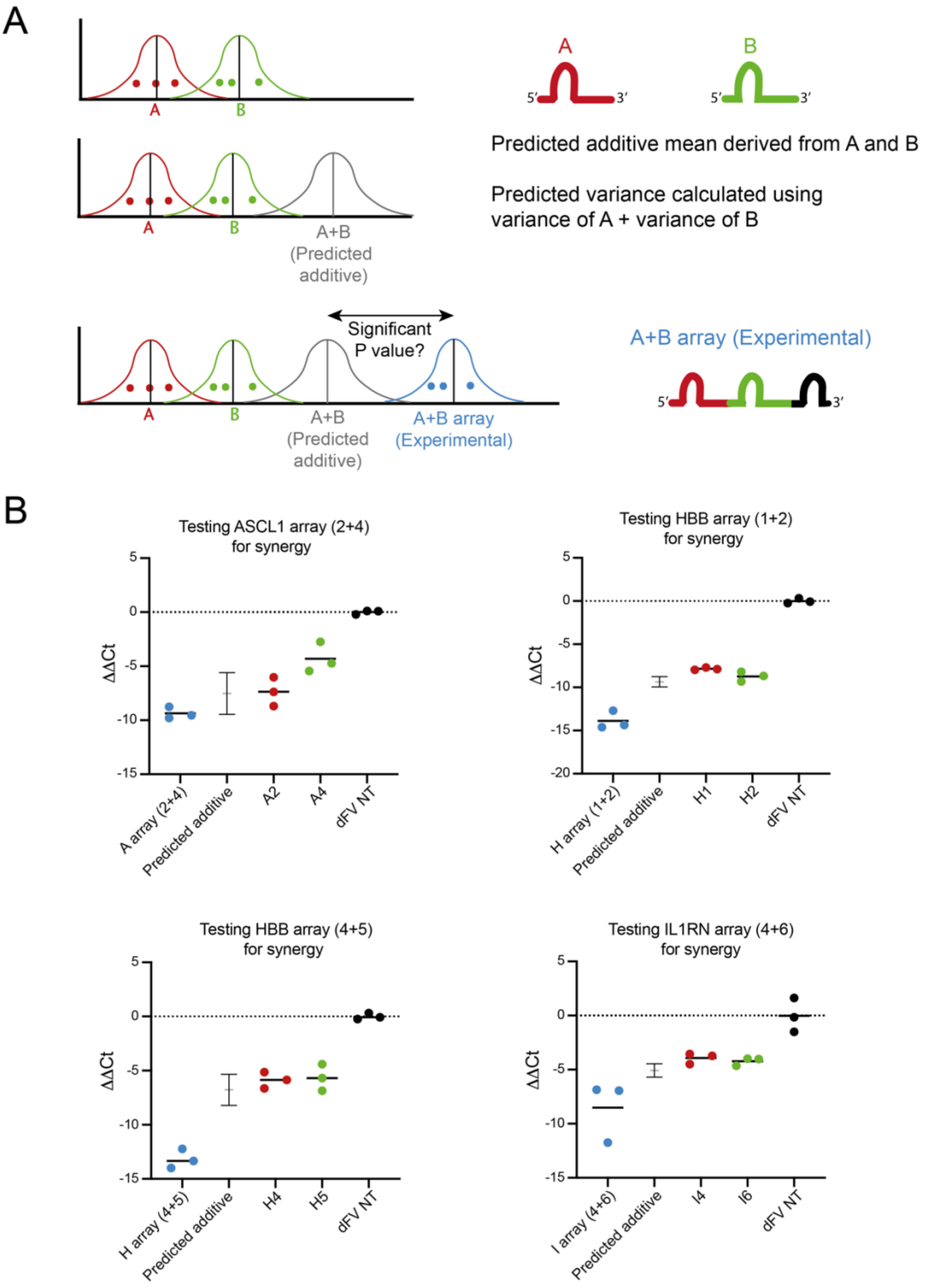

Supplementary Figure 2 – Testing crRNA arrays for synergistic transactivation
A) Schematic showing the approach used to evaluate whether synergistic activation of a target gene is observed. B) The ΔΔCT values used to calculate the relative mRNA expression shown in figure 5B were used to calculate a hypothetical additive distribution using the approach outlined in supplementary Note 1. The error bars denote the predicted standard deviation and the grey bar in the centre denotes the predicted mean. A one tailed t-test was then performed between each array and the associated hypothetical additive condition to test for synergistic (greater than additive) transactivation.

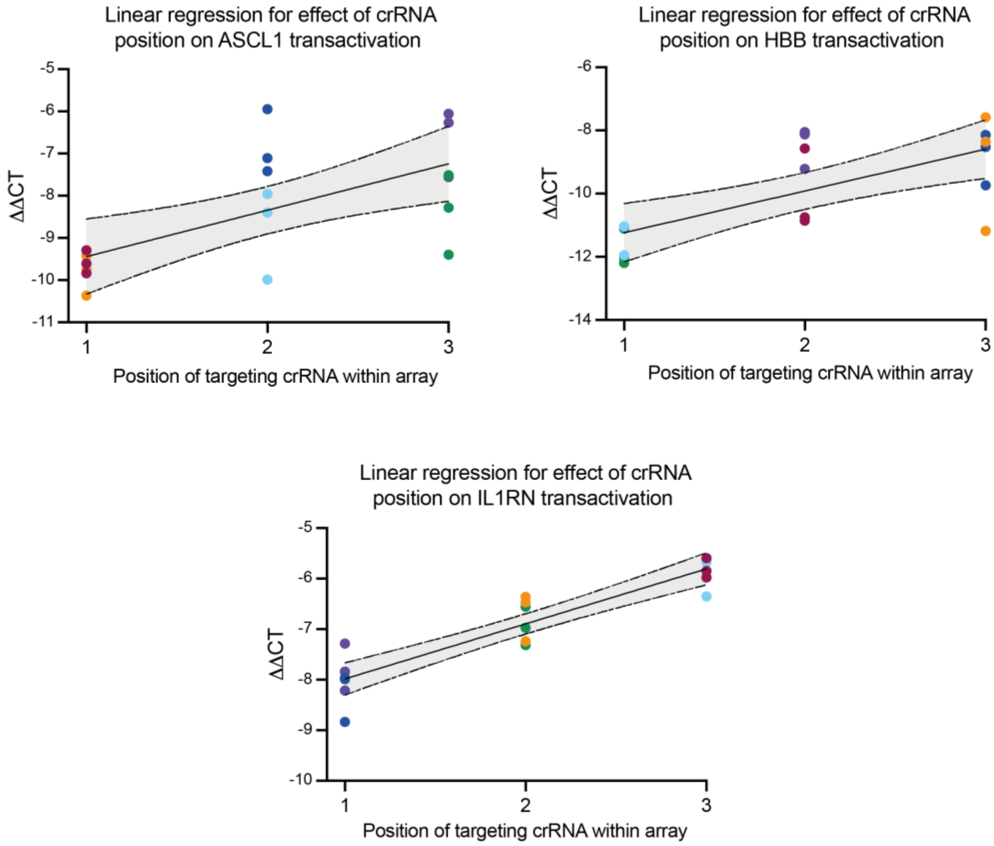

Supplementary Figure 3 – Simple linear regression for multiplexing crRNA array
The ΔΔCT values used to calculate the relative mRNA expression shown in figure 6 have been tested for a linear trend using linear regression analysis. For each of the three targeted genes, a significant increase in ΔΔCT (representing a decrease in relative mRNA abundance) is observed as you move the targeting crRNA pair from position 1 (most 5') towards position 3 (most 3') within the crRNA array (R^2 = 0.4160, P = 0.0039 for *ASCL1*; R^2 = 0.4876, P = 0.0013 for HBB and R^2 = 0.8457, P < 0.0001 for IL1RN.

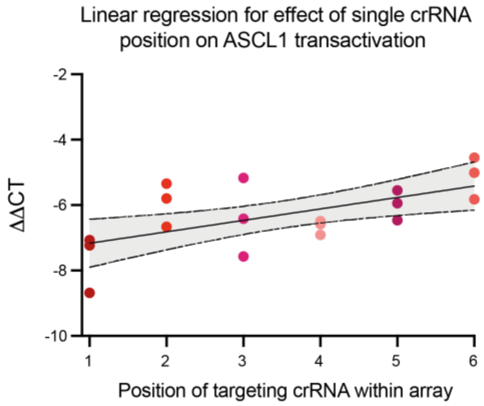

Supplementary Figure 4 – Simple linear regression for single active crRNA within array
The ΔΔCT values used to calculate the relative mRNA expression shown in figure 7 have been tested for a linear trend using linear regression analysis. A significant increase in ΔΔCT (representing a decrease in relative mRNA abundance) is observed as you move the targeting crRNA from position 1 (most 5') to position 6 (most 3') (R^2 = 0.3671, P = 0.0077).

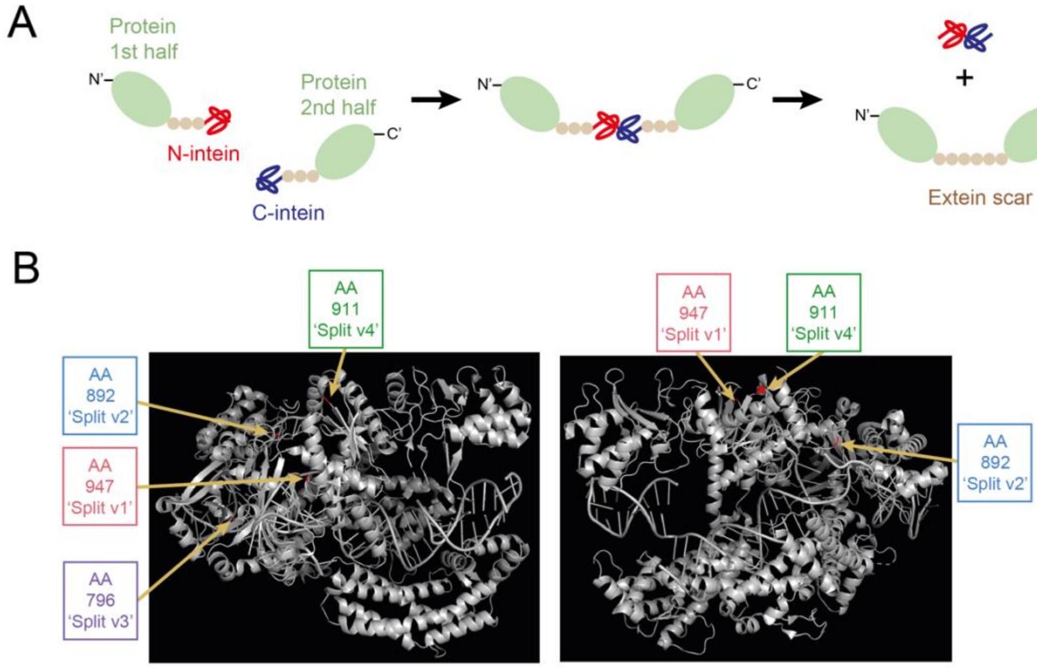

Supplementary Figure 5 – Designing split intein dFnCas12a-VPR versions
A) Schematic showing the mechanism of action for split inteins. When the first half of a protein is tagged on the C-terminus with an N-intein and the second half of the protein is tagged on the N-terminus with a C-intein then the two split inteins can interact and excise themselves from the protein, combining the two halves. This leaves a minimal extein scar within the recombined protein. B) Protein structure for dFnCas12a displayed using PyMol software, with the split intein target locations highlighted in red. The labels denote the amino acid number of each of the corresponding split sites.

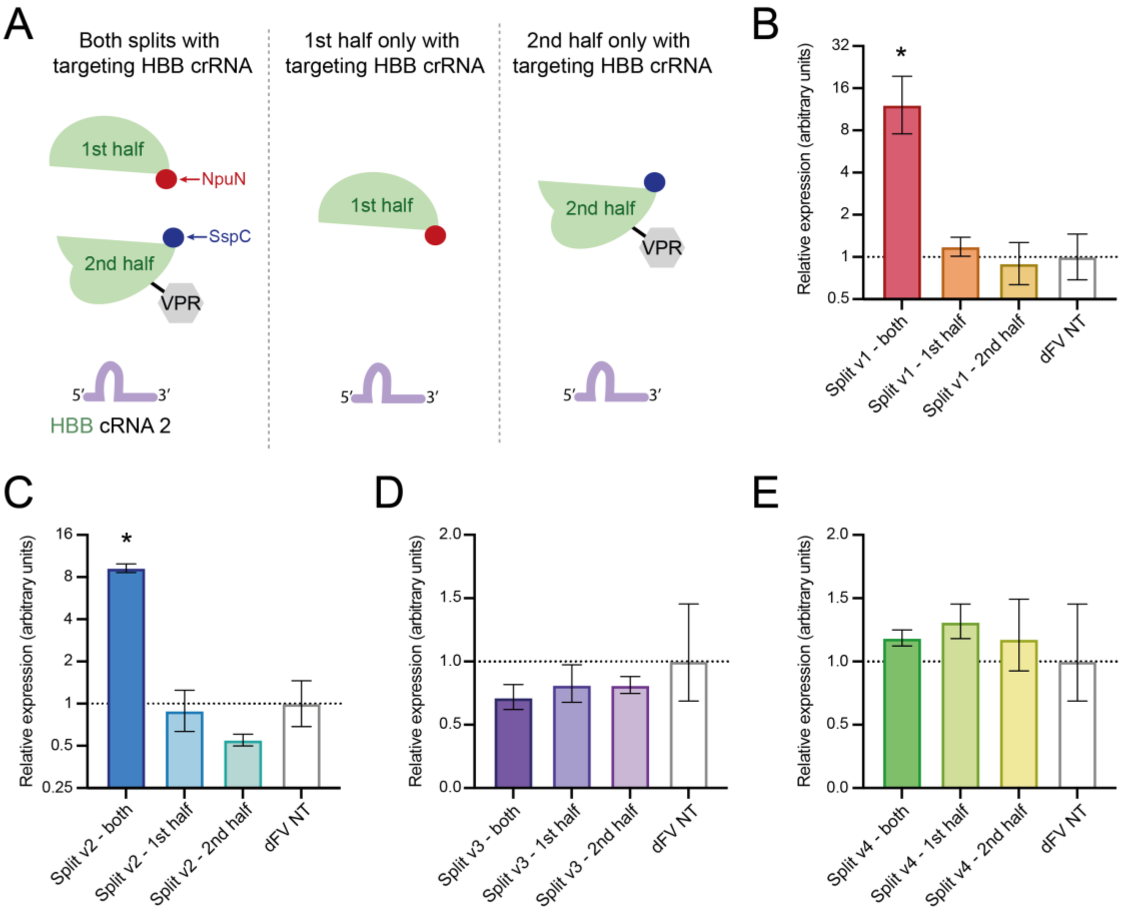

Supplementary Figure 6 – Testing split intein dFnCas12a-VPR for transactivation
A) Schematic showing the conditions tested for each of the four split-intein versions, with both halves or each half individually being delivered alongside the HBB crRNA 2. B-E) qRT-PCR results showing the relative expression of HBB, looking at both or each half delivered of each split-intein version, normalised to the non-targeting crRNA condition serving as a negative control. Results are from three biological replicates, error bars show SEM and stars (*) show results with a P value < 0.05 based on a Dunnett's multiple comparisons test.

SUPPLEMENTARY NOTE

Supplementary Note 1

The means and standard deviations for the predicted additive ΔΔCT distributions shown in supplementary figure 1B were derived by inferring the expected additive distribution for the fold change of mRNA when both targeting crRNA were present. The inferred mean ΔΔCT values was calculated by adding the geometric means for each of the two individual crRNA mRNA fold changes. The inferred variance was calculated by adding the variances for the CT values for each of the individual crRNA. From this variance the standard deviation was calculated by taking the square root of this value.

SUPPLEMENTARY TABLES

Supplementary table 1 –
Table including sequences for targeting gRNAs and crRNAs (targeting spacer sequence is coloured in blue).

Name	Sequence	Purpose
**Luc gRNA**	AGTCGCGTGTAGCGAAGCAgttttagagctaGAAAtagcaagttaaaataaggctagt ccgttatcaacttgaaaaagtggcaccgagtcggtgc	dCas9 gRNA for targeting Firefly luciferase plasmid
**As Luc crRNA**	TAATTTCTACTCTTGTAGATAGGCTAGCCATGCTTCGCTA	As crRNA for targeting Firefly lucifers plasmid
**Fn Luc crRNA**	TAATTTCTACTGTTGTAGATAGGCTAGCCATGCTTCGCTA	Fn crRNA for targeting Firefly lucifers plasmid
**Lb Luc crRNA**	TAATTTCTACTAAGTGTAGATAGGCTAGCCATGCTTCGCTA	Lb crRNA for targeting Firefly lucifers plasmid
**ASCL1 crRNA 1**	TAATTTCTACTGTTGTAGATAGCTGGGTTTGTTGTTGCAG	Targeting ASCL1 promoter
**ASCL1 crRNA 2**	TAATTTCTACTGTTGTAGATCAAGGAGcgggagaaaggaa	Targeting ASCL1 promoter
**ASCL1 crRNA 3**	TAATTTCTACTGTTGTAGATgggagtgggtgggaggaaga	Targeting ASCL1 promoter
**ASCL1 crRNA 4**	TAATTTCTACTGTTGTAGATTTGTTGCAGTGCGTGCGCCT	Targeting ASCL1 promoter
**ASCL1 crRNA 5**	TAATTTCTACTGTTGTAGATtcccgCTCCTTGCAAACTCT	Targeting ASCL1 promoter
**ASCL1 crRNA 6**	TAATTTCTACTGTTGTAGATctttctcccgCTCCTTGCAA	Targeting ASCL1 promoter
**HBB crRNA 1**	TAATTTCTACTGTTGTAGATTACTGATGGTATGGGGCCAA	Targeting HBB promoter
**HBB crRNA 2**	TAATTTCTACTGTTGTAGATAAGTCCAACTCCTAAGCCAG	Targeting HBB promoter
**HBB crRNA 3**	TAATTTCTACTGTTGTAGATCAAGTGTATTTACGTAATAT	Targeting HBB promoter
**HBB crRNA 4**	TAATTTCTACTGTTGTAGATGTAGCAATTTGTACTGATGG	Targeting HBB promoter
**HBB crRNA 5**	TAATTTCTACTGTTGTAGATGAGGGAGGGCTGAGGGTTTG	Targeting HBB promoter
**HBB crRNA 6**	TAATTTCTACTGTTGTAGATGGAGTTGGACTTCAAACCCTC	Targeting HBB promoter
**IL1RN crRNA 1**	TAATTTCTACTGTTGTAGATCGCAGATAAGAACCAGTTTG	Targeting IL1RN promoter
**IL1RN crRNA 2**	TAATTTCTACTGTTGTAGATCAGGAGGGTGACTCAGGCTA	Targeting IL1RN promoter
**IL1RN crRNA 3**	TAATTTCTACTGTTGTAGATGCATCAAGTCAGCCATCAGC	Targeting IL1RN promoter
**IL1RN crRNA 4**	TAATTTCTACTGTTGTAGATTCTGCATGTGACCTCCCATC	Targeting IL1RN promoter
**IL1RN crRNA 5**	TAATTTCTACTGTTGTAGATGTTTCTGCTAGCCTGAGTCA	Targeting IL1RN promoter
**IL1RN crRNA 6**	TAATTTCTACTGTTGTAGATGCCAGCATGAGGAGATGGGC	Targeting IL1RN promoter

Supplementary table 2 –
Table including primers for qRT-PCR analysis

Name	Sequence
**HBB qPCR F**	AAG CTG CAC GTG GAT CCT GA
**HBB qPCR R**	ATT AGC CAC ACC AGC CAC CA
**ASCL1 qPCR F**	CGCGGCCAACAAGAAGATG
**ASCL1 qPCR R**	CGACGAGTAGGATGAGACCG
**IL1RN qPCR F**	GGAATCCATGGAGGGAAGAT
**IL1RN qPCR R**	TGTTCTCGCTCAGGTCAGTG
**RCN1 qPCR F (normalising gene)**	CCCGTAATTGGTAGCTTGGC
**RCN1 qPCR R (normalising gene)**	TGAGATTCGCCACTGGATCC

Supplementary table 3 –
Table including FnCas12a derived synthetic transcription factors (full length and split coding sequences).

Name	Sequence
**dFnCas12a-VPR**	atgagcatctaccaggagttcgtcaacaagtattcactgagtaagacactgcggttcgagctgatcccacagggcaagacactggagaacatcaaggc<br>ccgaggcctgattctggacgatgagaagcgggcaaaagactataagaaagccaagcagatcattgataaataccaccagttctttatcgaggaaattct<br>gagctccgtgtgcatcagtgaggatctgctgcagaattactcagacgtgtacttcaagctgaagaagagcgacgatgacaacctgcagaaggacttcaa<br>gtccgccaaggacaccatcaagaaacagattagcgagtacatcaaggactccgaaaagtttaaaaatctgttcaaccagaatctgatcgatgctaaga<br>aaggccaggagtccgacctgatcctgtggctgaaacagtctaaggacaatgggattgaactgttcaaggctaactccgatatcactgatattgacgaggc<br>actggaaatcatcaagagcttcaagggatggaccacatactttaaaggcttccacgagaaccgcaagaacgtgtactccagcaacgacattcctacctc<br>catcatctaccgaatcgtcgatgacaatctgccaaagttcctggagaacaaggccaaatatgaatctctgaaggacaaagctcccgaggcaattaattac<br>gaacagatcaagaaagatctggctgaggaactgacattcgatatcgactataagactagcgaggtgaaccagagggtcttttccctggacgaggtgtttg<br>aaatcgccaatttcaacaattacctgaaccagtccggcattactaaattcaataccatcattggcgggaagtttgtgaacggggagaataccaagcgcaa<br>gggaattaacgaatacatcaatctgtatagccagcagatcaacgacaaaactctgaagaaatacaagatgtctgtgctgttcaaacagatcctgagtgat<br>accgagtccaagtcttttgtcattgataaactggaagatgactcagacgtggtcactaccatgcagagcttttatgagcagatcgccgctttcaagacagtgg<br>aggaaaaatctattaaggaaactctgagtctgctgttcgatgacctgaaagcccagaagctggacctgagtaagatctacttcaaaaacgataagagtct<br>gacagacctgtcacagcaggtgtttgatgactattccgtgattgggaccgccgtcctggagtacattacacagcagatcgctccaaagaacctggataatc<br>cctctaagaaagagcaggaactgatcgctaagaaaaccgagaaggcaaaatatctgagtctggaaacaattaagctggcactggaggagttcaacaa<br>gcacagggatattgacaaacagtgccgctttgaggaaatcctggccaacttcgcagccatccccatgatttttgatgagatcgcccagaacaaagacaat<br>ctggctcagatcagtattaagtaccagaaccagggcaagaaagacctgctgcaggcttcagcagaagatgacgtgaaagccatcaaggatctgctgg<br>accagaccaacaatctgctgcacaagctgaaaatcttccatattagtcagtcagaggataaggctaatatcctggataaagacgaacacttctacctggtg<br>ttcgaggaatgttacttcgagctggcaaacattgtcccoctgtataacaagattaggaactacatcacacagaagccttactctgacgagaagtttaaactg<br>aacttcgaaaatagtaccctggccaacgggtgggataagaacaaggagcctgacaacacagctatcctgttcatcaaggatgacaagtactatctggg<br>agtgatgaataagaaaaacaataagatcttcgatgacaaagccattaaggagaacaaaggggaaggatacaagaaaatcgtgtataagctgctgccc<br>ggcgcaaataagatgctgcctaaggtgttcttcagcgccaagagtatcaaattctacaacccatccgaggacatcctgcggattagaaatcactcaacac<br>atactaagaacgggagcccccagaagggatatgagaaatttgagttcaacatcgaggattgcaggaagtttattgacttctacaagcagagcatctccaa<br>acaccctgaatggaaggattttggcttccggttttccgacacacagagatataactctatcgacgagttctaccgcgaggtggaaaatcaggggtataagc<br>tgacttttgagaacatttctgaaagttacatcgacagcgtggtcaatcagggaaagctgtacctgttccagatctataacaaagatttttcagcatacagcaa<br>gggcagaccaaacctgcatacactgtactggaaggccctgttcgatgagaggaatctgcaggacgtggtctataaactgaacggagaggccgaactgt<br>tttaccggaagcagtctattcctaagaaaatcactcacccagctaaggaggccatcgctaacaagaacaaggacaatcctaagaaagagagcgtgttc<br>gaatacgatctgattaaggacaagcggttcaccgaagataagttctttttccattgtccaatcaccattaacttcaagtcaagcggcgctaacaagttcaacg<br>acgagatcaatctgctgctgaaggaaaaagcaaacgatgtgcacatcctgagcattgcgcgaggagagcggcatctggcctactataccctggtggatg<br>gcaaagggaatatcattaagcaggatacattcaacatcattggcaatgaccggatgaaaaccaactaccacgataaactggctgcaatcgagaaggat<br>agagactcagctaggaaggactggaagaaaatcaacaacattaaggagatgaaggaaggctatctgagccaggtggtccatgagattgcaaagctg<br>gtcatcgaatacaatgccattgtggtgttcgaggatctgaacttcggctttaagagggggcgctttaaggtggaaaaacaggtctatcagaagctggagaa<br>aatgctgatcgaaaagctgaattacctggtgtttaaagataacgagttcgacaagaccggaggcgtcctgagagcctaccagctgacagctccctttgaa<br>actttcaagaaaatgggaaaacagacaggcatcatctactatgtgccagccggattcacttccaagatctgccccgtgaccggctttgtcaaccagctgta<br>ccctaaatatgagtcagtgagcaagtcccaggaatttttcagcaagttcgataagatctgttataatctggacaaggggtacttcgagttttccttcgattacaa<br>gaacttcggcgacaaggccgctaaggggaaatggaccattgcctccttcggatctcgcctgatcaactttcgaaattccgataaaaaccacaattgggac<br>actagggaggtgtacccaaccaaggagctggaaaagctgctgaaagactactctatcgagtatggacatggcgaatgcatcaaggcagccatctgtgg<br>cgagagtgataagaaatttttcgccaagctgacctcagtgctgaatacaatcctgcagatgcggaactcaaagaccgggacagaactggactatctgatt<br>agccccgtggctgatgtcaacggaaacttcttcgacagcagacaggcacccaaaaatatgcctcaggatgcagacgccaacggggcctaccacatcg<br>ggctgaagggactgatgctgctgggccggatcaagaacaatcaggaggggaagaagctgaacctggtcattaagaacgaggaatacttcgagtttgtc<br>cagaatagaaataacaaaaggccggcggccacgaaaaaggccggccaggcaaaaaagaaaaaggGATCCGGAACCCGGGCTGA<br>CGCATTGGACGATTTTGATCTGGATATGCTGGGAAGTGACGCCCTCGATGATTTTGACCTTGACATGCTT<br>GGTTCGGATGCCCTTGATGACTTTGACCTCGACATGCTCGGCAGTGACGCCCTTGATGATTTCGACCTG<br>GACATGCTGATTAACTCTAGAAGTTCCGGATCTCCGAAAAAGAAACGCAAAGTTGGTAGCCAGTACCTG<br>CCCGACACCGACGACCGGCACCGGATCGAGGAAAAGCGGAAGCGGACCTACGAGACATTCAAGAGCAT<br>CATGAAGAAGTCCCCCTTCAGCGGCCCCACCGACCCTAGACCTCCACCTAGAAGAATCGCCGTGCCCA<br>GCAGATCCAGCGCCAGCGTGCCAAAACCTGCCCCCCAGCCTTACCCCTTCACCAGCAGCCTGAGCACC

	ATCAACTACGACGAGTTCCCTACCATGGTGTTCCCCAGCGGCCAGATCTCTCAGGCCTCTGCTCTGGCT<br>CCAGCCCCTCCTCAGGTGCTGCCTCAGGCTCCTGCTCCTGCACCAGCTCCAGCCATGGTGTCTGCACT<br>GGCTCAGGCACCAGCACCCGTGCCTGTGCTGGCTCCTGGACCTCCACAGGCTGTGGCTCCACCAGCCC<br>CTAAACCTACACAGGCCGGCGAGGGCACACTGTCTGAAGCTCTGCTGCAGCTGCAGTTCGACGACGAG<br>GATCTGGGAGCCCTGCTGGGAAACAGCACCGATCCTGCCGTGTTCACCGACCTGGCCAGCGTGGACAA<br>CAGCGAGTTCCAGCAGCTGCTGAACCAGGGCATCCCTGTGGCCCCTCACACCACCGAGCCCATGCTGA<br>TGGAATACCCCGAGGCCATCACCCGGCTCGTGACAGGCGCTCAGAGGCCTCCTGATCCAGCTCCTGCC<br>CCTCTGGGAGCACCAGGCCTGCCTAATGGACTGCTGTCTGGCGACGAGGACTTCAGCTCTATCGCCGA<br>TATGGATTTCTCAGCCTTGCTGGGCTCTGGCAGCGGCAGCCGGGATTCCAGGGAAGGGATGTTTTTGC<br>CGAAGCCTGAGGCCGGCTCCGCTATTAGTGACGTGTTTGAGGGCCGCGAGGTGTGCCAGCCAAAACGA<br>ATCCGGCCATTTCATCCTCCAGGAAGTCCATGGGCCAACCGCCCACTCCCCGCCAGCCTCGCACCAAC<br>ACCAACCGGTCCAGTACATGAGCCAGTCGGGTCACTGACCCCGGCACCAGTCCCTCAGCCACTGGATC<br>CAGCGCCCGCAGTGACTCCCGAGGCCAGTCACCTGTTGGAGGATCCCGATGAAGAGACGAGCCAGGC<br>TGTCAAAGCCCTTCGGGAGATGGCCGATACTGTGATTCCCCAGAAGGAAGAGGCTGCAATCTGTGGCC<br>AAATGGACCTTTCCCATCCGCCCCCAAGGGGCCATCTGGATGAGCTGACAACCACACTTGAGTCCATGA<br>CCGAGGATCTGAACCTGGACTCACCCCTGACCCCGGAATTGAACGAGATTCTGGATACCTTCCTGAACG<br>ACGAGTGCCTCTTGCATGCCATGCATATCAGCACAGGACTGTCCATCTTCGACACATCTCTGTTTTCCGG<br>ATGA
**split v1-NpuN half1**	ATGgagcagaaactcattagtgaggaagatctgATGAGCATCTACCAGGAGTTCGTCAACAAGtattcactgagtaagacactgc<br>ggttcgagctgatcccacagggcaagacactggagaacatcaaggcccgaggcctgattctggacgatgagaagcgggcaaaagactataagaaa<br>gccaagcagatcattgataaataccaccagttctttatcgaggaaattctgagctccgtgtgcatcagtgaggatctgctgcagaattactcagacgtgtactt<br>caagctgaagaagagcgacgatgacaacctgcagaaggacttcaagtccgccaaggacaccatcaagaaacagattagcgagtacatcaaggact<br>ccgaaaagtttaaaaatctgttcaaccagaatctgatcgatgctaagaaaggccaggagtccgacctgatcctgtggctgaaacagtctaaggacaatg<br>ggattgaactgttcaaggctaactccgatatcactgatattgacgaggcactggaaatcatcaagagcttcaagggatggaccacatactttaaaggcttc<br>cacgagaaccgcaagaacgtgtactccagcaacgacattcctacctccatcatctaccgaatcgtcgatgacaatctgccaaagttcctggagaacaag<br>gccaaatatgaatctctgaaggacaaagctcccgaggcaattaattacgaacagatcaagaaagatctggctgaggaactgacattcgatatcgactat<br>aagactagcgaggtgaaccagagggtcttttccctggacgaggtgtttgaaatcgccaatttcaacaattacctgaaccagtccggcattactaaattcaat<br>accatcattggcgggaagtttgtgaacggggagaataccaagcgcaagggaattaacgaatacatcaatctgtatagccagcagatcaacgacaaaa<br>ctctgaagaaatacaagatgtctgtgctgttcaaacagatcctgagtgataccgagtccaagtcttttgtcattgataaactggaagatgactcagacgtggt<br>cactaccatgcagagcttttatgagcagatcgccgctttcaagacagtggaggaaaaatctattaaggaaactctgagtctgctgttcgatgacctgaaag<br>cccagaagctggacctgagtaagatctacttcaaaaacgataagagtctgacagacctgtcacagcaggtgtttgatgactattccgtgattgggaccgcc<br>gtcctggagtacattacacagcagatcgctccaaagaacctggataatccctctaagaaagagcaggaactgatcgctaagaaaaccgagaaggcaa<br>aatatctgagtctggaaacaattaagctggcactggaggagttcaacaagcacagggatattgacaaacagtgccgctttgaggaaatcctggccaactt<br>cgcagccatccccatgatttttgatgagatcgcccagaacaaagacaatctggctcagatcagtattaagtaccagaaccagggcaagaaagacctgct<br>gcaggcttcagcagaagatgacgtgaaagccatcaaggatctgctggaccagaccaacaatctgctgcacaagctgaaaatcttccatattagtcagtc<br>agaggataaggctaatatcctggataaagacgaacacttctacctggtgttcgaggaatgttacttcgagctggcaaacattgtcccccctgtataacaagat<br>taggaactacatcacacagaagccttactctgacgagaagtttaaactgaacttcgaaaatagtaccctggccaacgggtgggataagaacaaggagc<br>ctgacaacacagctatcctgttcatcaaggatgacaagtactatctgggagtgatgaataagaaaaacaataagatcttcgatgacaaagccattaagga<br>gaacaaaggggaaggatacaagaaaatcgtgtataagctgctgcccggcgcaaataagatgctgcctaaggtgttcttcagcgccaagagtatcaaatt<br>ctacaacccatccgaggacatcctgcggattagaaatcactcaacacatactaagaacgggagcccccagaagggatatgagaaatttgagttcaaca<br>tcgaggattgcaggaagtttattgacttctacaagcagagcatctccaaacaccctgaatggaaggattttggcttccggttttccgacacacagagatata<br>actctatcgacgagttctaccgcgaggtggaaaatcaggggtataagctgacttttgagaacatttctgaaagttacatcgacagcgtggtcaatcaggga<br>aagctgtacctgttccagatctataacaaagatttttcagcatacagcaagggcagaccaaacctgcatacactgtactggaaggccctgttcgatgagag<br>gaatctgcaggacgtggtctataaactgaacggagaggccgaactgttttaccggaagcagtctattcctaagaaaatcactcacccagctaaggaggc<br>catcgctaacaagaacaaggacaatcctaagaaagagagcgtgttcgaatacgatctgattaaggacaagcggttcaccgaagataagttctttttccatt<br>gtccaatcaccattaacttcaagtcaagcggcgctaacaagttcaacgacgagatcaatctgctgctgaaggaaaaagcaaacgatgtgcacatcctga<br>gcattgcgcgaggagagcggcatctggcctactataccctggtggatggcaaagggaatatcattaagcaggatacattcaacatcattggcaatgacT<br>GCCTTTCCTACGAAACCGAGATCCTGACAGTGGAGTACGGGTCACTGCCAATTGGCAAGATTGTTGAGA<br>AAAGGATAGAATGCACTGTGTACAGCGTGGATAATAACGGTAACATTTATACCCAGCCTGTGGCACAGT<br>GGCATGATCGCGGGGAACAGGAGGTTTTTGAATACTGTTTGGAGGACGGATCTCTCATACGTGCCACAA<br>AAGACCATAAATTCATGACCGTCGACGGTCAGATGCTGCCTATCGACGAAATTTTTGAGCGGGAACTCG<br>ACCTTATGCGGGTTGACAATCTGCCCAACACCTCATGA
**split v2-NpuN half1**	ATGgagcagaaactcattagtgaggaagatctgATGAGCATCTACCAGGAGTTCGTCAACAAGtattcactgagtaagacactgc<br>ggttcgagctgatcccacagggcaagacactggagaacatcaaggcccgaggcctgattctggacgatgagaagcgggcaaaagactataagaaa<br>gccaagcagatcattgataaataccaccagttctttatcgaggaaattctgagctccgtgtgcatcagtgaggatctgctgcagaattactcagacgtgtactt<br>caagctgaagaagagcgacgatgacaacctgcagaaggacttcaagtccgccaaggacaccatcaagaaacagattagcgagtacatcaaggact<br>ccgaaaagtttaaaaatctgttcaaccagaatctgatcgatgctaagaaaggccaggagtccgacctgatcctgtggctgaaacagtctaaggacaatg<br>ggattgaactgttcaaggctaactccgatatcactgatattgacgaggcactggaaatcatcaagagcttcaagggatggaccacatactttaaaggcttc<br>cacgagaaccgcaagaacgtgtactccagcaacgacattcctacctccatcatctaccgaatcgtcgatgacaatctgccaaagttcctggagaacaag<br>gccaaatatgaatctctgaaggacaaagctcccgaggcaattaattacgaacagatcaagaaagatctggctgaggaactgacattcgatatcgactat<br>aagactagcgaggtgaaccagagggtcttttccctggacgaggtgtttgaaatcgccaatttcaacaattacctgaaccagtccggcattactaaattcaat<br>accatcattggcgggaagtttgtgaacggggagaataccaagcgcaagggaattaacgaatacatcaatctgtatagccagcagatcaacgacaaaa<br>ctctgaagaaatacaagatgtctgtgctgttcaaacagatcctgagtgataccgagtccaagtcttttgtcattgataaactggaagatgactcagacgtggt<br>cactaccatgcagagcttttatgagcagatcgccgctttcaagacagtggaggaaaaatctattaaggaaactctgagtctgctgttcgatgacctgaaag<br>cccagaagctggacctgagtaagatctacttcaaaaacgataagagtctgacagacctgtcacagcaggtgtttgatgactattccgtgattgggaccgcc<br>gtcctggagtacattacacagcagatcgctccaaagaacctggataatccctctaagaaagagcaggaactgatcgctaagaaaaccgagaaggcaa<br>aatatctgagtctggaaacaattaagctggcactggaggagttcaacaagcacagggatattgacaaacagtgccgctttgaggaaatcctggccaactt<br>cgcagccatccccatgatttttgatgagatcgcccagaacaaagacaatctggctcagatcagtattaagtaccagaaccagggcaagaaagacctgct<br>gcaggcttcagcagaagatgacgtgaaagccatcaaggatctgctggaccagaccaacaatctgctgcacaagctgaaaatcttccatattagtcagtc<br>agaggataaggctaatatcctggataaagacgaacacttctacctggtgttcgaggaatgttacttcgagctggcaaacattgtcccccctgtataacaagat<br>taggaactacatcacacagaagccttactctgacgagaagtttaaactgaacttcgaaaatagtaccctggccaacgggtgggataagaacaaggagc<br>ctgacaacacagctatcctgttcatcaaggatgacaagtactatctgggagtgatgaataagaaaaacaataagatcttcgatgacaaagccattaagga<br>gaacaaaggggaaggatacaagaaaatcgtgtataagctgctgcccggcgcaaataagatgctgcctaaggtgttcttcagcgccaagagtatcaaatt

	ctacaacccatccgaggacatcctgcggattagaaatcactcaacacatactaagaacgggagcccccagaagggatatgagaaatttgagttcaaca<br>tcgaggattgcaggaagtttattgacttctacaagcagagcatctccaaacaccctgaatggaaggattttggcttccggttttccgacacacagagatata<br>actctatcgacgagttctaccgcgaggtggaaaatcaggggtataagctgacttttgagaacatttctgaaagttacatcgacagcgtggtcaatcaggga<br>aagctgtacctgttccagatctataacaaagatttttcagcatacagcaagggcagaccaaacctgcatacactgtactggaaggccctgttcgatgagag<br>gaatctgcaggacgtggtctataaactgaacggagaggccgaactgttttaccggaagcagtctattcctaagaaaatcactcacccagctaaggaggc<br>catcgctaacaagaacaaggacaatcctaagaaagagagcgtgttcgaatacgatctgattaaggacaagcggttcaccgaagataagttctttttccatt<br>gtccaatcaccattaacttcaagtcaagcggcTGCCTTTCCTACGAAACCGAGATCCTGACAGTGGAGTACGGGTCACT<br>GCCAATTGGCAAGATTGTTGAGAAAAGGATAGAATGCACTGTGTACAGCGTGGATAATAACGGTAACATT<br>TATACCCAGCCTGTGGCACAGTGGCATGATCGCGGGGAACAGGAGGTTTTTGAATACTGTTTGGAGGAC<br>GGATCTCTCATACGTGCCACAAAAGACCATAAATTCATGACCGTCGACGGTCAGATGCTGCCTATCGAC<br>GAAATTTTTGAGCGGGAACTCGACCTTATGCGGGTTGACAATCTGCCCAACACCTCATGA
**split v3-NpuN half1**	ATGgagcagaaactcattagtgaggaagatctgATGAGCATCTACCAGGAGTTCGTCAACAAGtattcactgagtaagacactgc<br>ggttcgagctgatcccacagggcaagacactggagaacatcaaggcccgaggcctgattctggacgatgagaagcgggcaaaagactataagaaa<br>gccaagcagatcattgataaataccaccagttctttatcgaggaaattctgagctccgtgtgcatcagtgaggatctgctgcagaattactcagacgtgtactt<br>caagctgaagaagagcgacgatgacaacctgcagaaggacttcaagtccgccaaggacaccatcaagaaacagattagcgagtacatcaaggact<br>ccgaaaagtttaaaaatctgttcaaccagaatctgatcgatgctaagaaaggccaggagtccgacctgatcctgtggctgaaacagtctaaggacaatg<br>ggattgaactgttcaaggctaactccgatatcactgatattgacgaggcactggaaatcatcaagagcttcaagggatggaccacatactttaaaggcttc<br>cacgagaaccgcaagaacgtgtactccagcaacgacattcctacctccatcatctaccgaatcgtcgatgacaatctgccaaagttcctggagaacaag<br>gccaaatatgaatctctgaaggacaaagctcccgaggcaattaattacgaacagatcaagaaagatctggctgaggaactgacattcgatatcgactat<br>aagactagcgaggtgaaccagagggtcttttccctggacgaggtgtttgaaatcgccaatttcaacaattacctgaaccagtccggcattactaaattcaat<br>accatcattggcgggaagtttgtgaacggggagaataccaagcgcaagggaattaacgaatacatcaatctgtatagccagcagatcaacgacaaaa<br>ctctgaagaaatacaagatgtctgtgctgttcaaacagatcctgagtgataccgagtccaagtcttttgtcattgataaactggaagatgactcagacgtggt<br>cactaccatgcagagcttttatgagcagatcgccgctttcaagacagtggaggaaaaatctattaaggaaactctgagtctgctgttcgatgacctgaaag<br>cccagaagctggacctgagtaagatctacttcaaaaacgataagagtctgacagacctgtcacagcaggtgtttgatgactattccgtgattgggaccgcc<br>gtcctggagtacattacacagcagatcgctccaaagaacctggataatccctctaagaaagagcaggaactgatcgctaagaaaaccgagaaggcaa<br>aatatctgagtctggaaacaattaagctggcactggaggagttcaacaagcacagggatattgacaaacagtgccgctttgaggaaatcctggccaactt<br>cgcagccatccccatgatttttgatgagatcgcccagaacaaagacaatctggctcagatcagtattaagtaccagaaccagggcaagaaagacctgct<br>gcaggcttcagcagaagatgacgtgaaagccatcaaggatctgctggaccagaccaacaatctgctgcacaagctgaaaatcttccatattagtcagtc<br>agaggataaggctaatatcctggataaagacgaacacttctacctggtgttcgaggaatgttacttcgagctggcaaacattgtcccсctgtataacaagat<br>taggaactacatcacacagaagccttactctgacgagaagtttaaactgaacttcgaaaatagtaccctggccaacgggtgggataagaacaaggagc<br>ctgacaacacagctatcctgttcatcaaggatgacaagtactatctgggagtgatgaataagaaaaacaataagatcttcgatgacaaagccattaagga<br>gaacaaaggggaaggatacaagaaaatcgtgtataagctgctgcccggcgcaaataagatgctgcctaaggtgttcttcagcgccaagagtatcaaatt<br>ctacaacccatccgaggacatcctgcggattagaaatcactcaacacatactaagaacgggagcccccagaagggatatgagaaatttgagttcaaca<br>tcgaggattgcaggaagtttattgacttctacaagcagagcatctccaaacaccctgaatggaaggattttggcttccggttttccgacacacagagatata<br>actctatcgacgagttctaccgcgaggtggaaaatcaggggtataagctgacttttgagaacatttctgaaagttacatcgacagcgtggtcaatcaggga<br>aagctgtacctgttccagatctataacaaagatttttcagcatacTGCCTTTCCTACGAAACCGAGATCCTGACAGTGGAGTACG<br>GGTCACTGCCAATTGGCAAGATTGTTGAGAAAAGGATAGAATGCACTGTGTACAGCGTGGATAATAACG<br>GTAACATTTATACCCAGCCTGTGGCACAGTGGCATGATCGCGGGGAACAGGAGGTTTTTGAATACTGTT<br>TGGAGGACGGATCTCTCATACGTGCCACAAAAGACCATAAATTCATGACCGTCGACGGTCAGATGCTGC<br>CTATCGACGAAATTTTTGAGCGGGAACTCGACCTTATGCGGGTTGACAATCTGCCCAACACCTCATGA
**split v4-NpuN half1**	ATGgagcagaaactcattagtgaggaagatctgATGAGCATCTACCAGGAGTTCGTCAACAAGtattcactgagtaagacactgc<br>ggttcgagctgatcccacagggcaagacactggagaacatcaaggcccgaggcctgattctggacgatgagaagcgggcaaaagactataagaaa<br>gccaagcagatcattgataaataccaccagttctttatcgaggaaattctgagctccgtgtgcatcagtgaggatctgctgcagaattactcagacgtgtactt<br>caagctgaagaagagcgacgatgacaacctgcagaaggacttcaagtccgccaaggacaccatcaagaaacagattagcgagtacatcaaggact<br>ccgaaaagtttaaaaatctgttcaaccagaatctgatcgatgctaagaaaggccaggagtccgacctgatcctgtggctgaaacagtctaaggacaatg<br>ggattgaactgttcaaggctaactccgatatcactgatattgacgaggcactggaaatcatcaagagcttcaagggatggaccacatactttaaaggcttc<br>cacgagaaccgcaagaacgtgtactccagcaacgacattcctacctccatcatctaccgaatcgtcgatgacaatctgccaaagttcctggagaacaag<br>gccaaatatgaatctctgaaggacaaagctcccgaggcaattaattacgaacagatcaagaaagatctggctgaggaactgacattcgatatcgactat<br>aagactagcgaggtgaaccagagggtcttttccctggacgaggtgtttgaaatcgccaatttcaacaattacctgaaccagtccggcattactaaattcaat<br>accatcattggcgggaagtttgtgaacggggagaataccaagcgcaagggaattaacgaatacatcaatctgtatagccagcagatcaacgacaaaa<br>ctctgaagaaatacaagatgtctgtgctgttcaaacagatcctgagtgataccgagtccaagtcttttgtcattgataaactggaagatgactcagacgtggt<br>cactaccatgcagagcttttatgagcagatcgccgctttcaagacagtggaggaaaaatctattaaggaaactctgagtctgctgttcgatgacctgaaag<br>cccagaagctggacctgagtaagatctacttcaaaaacgataagagtctgacagacctgtcacagcaggtgtttgatgactattccgtgattgggaccgcc<br>gtcctggagtacattacacagcagatcgctccaaagaacctggataatccctctaagaaagagcaggaactgatcgctaagaaaaccgagaaggcaa<br>aatatctgagtctggaaacaattaagctggcactggaggagttcaacaagcacagggatattgacaaacagtgccgctttgaggaaatcctggccaactt<br>cgcagccatccccatgatttttgatgagatcgcccagaacaaagacaatctggctcagatcagtattaagtaccagaaccagggcaagaaagacctgct<br>gcaggcttcagcagaagatgacgtgaaagccatcaaggatctgctggaccagaccaacaatctgctgcacaagctgaaaatcttccatattagtcagtc<br>agaggataaggctaatatcctggataaagacgaacacttctacctggtgttcgaggaatgttacttcgagctggcaaacattgtcccсctgtataacaagat<br>taggaactacatcacacagaagccttactctgacgagaagtttaaactgaacttcgaaaatagtaccctggccaacgggtgggataagaacaaggagc<br>ctgacaacacagctatcctgttcatcaaggatgacaagtactatctgggagtgatgaataagaaaaacaataagatcttcgatgacaaagccattaagga<br>gaacaaaggggaaggatacaagaaaatcgtgtataagctgctgcccggcgcaaataagatgctgcctaaggtgttcttcagcgccaagagtatcaaatt<br>ctacaacccatccgaggacatcctgcggattagaaatcactcaacacatactaagaacgggagcccccagaagggatatgagaaatttgagttcaaca<br>tcgaggattgcaggaagtttattgacttctacaagcagagcatctccaaacaccctgaatggaaggattttggcttccggttttccgacacacagagatata<br>actctatcgacgagttctaccgcgaggtggaaaatcaggggtataagctgacttttgagaacatttctgaaagttacatcgacagcgtggtcaatcaggga<br>aagctgtacctgttccagatctataacaaagatttttcagcatacagcaagggcagaccaaacctgcatacactgtactggaaggccctgttcgatgagag<br>gaatctgcaggacgtggtctataaactgaacggagaggccgaactgttttaccggaagcagtctattcctaagaaaatcactcacccagctaaggaggc<br>catcgctaacaagaacaaggacaatcctaagaaagagagcgtgttcgaatacgatctgattaaggacaagcggttcaccgaagataagttctttttccatt<br>gtccaatcaccattaacttcaagtcaagcggcgctaacaagttcaacgacgagatcaatctgctgctgaaggaaaaagcaaacgatgtgTGCCTTT<br>CCTACGAAACCGAGATCCTGACAGTGGAGTACGGGTCACTGCCAATTGGCAAGATTGTTGAGAAAAGGA<br>TAGAATGCACTGTGTACAGCGTGGATAATAACGGTAACATTTATACCCAGCCTGTGGCACAGTGGCATG<br>ATCGCGGGGAACAGGAGGTTTTTGAATACTGTTTGGAGGACGGATCTCTCATACGTGCCACAAAAGACC<br>ATAAATTCATGACCGTCGACGGTCAGATGCTGCCTATCGACGAAATTTTTGAGCGGGAACTCGACCTTAT<br>GCGGGTTGACAATCTGCCCAACACCTCATGA

**SspC half2-split v1**	ATGGTGAAAGTAATTGGGCGCCGGTCCCTGGGAGTCCAACGAATCTTTGACATTGGTCTGCGTCAAGAT<br>CATAATTTTCTTCTTGCTAATGGAGCTATCGCAGCAAACTGCTTTAATcggatgaaaaccaactaccacgataaactgg<br>ctgcaatcgagaaggatagagactcagctaggaaggactggaagaaaatcaacaacattaaggagatgaaggaaggctatctgagccaggtggtcc<br>atgagattgcaaagctggtcatcgaatacaatgccattgtggtgttcgaggatctgaacttcggctttaagagggggcgctttaaggtggaaaaacaggtct<br>atcagaagctggagaaaatgctgatcgaaaagctgaattacctggtgtttaaagataacgagttcgacaagaccggaggcgtcctgagagcctaccag<br>ctgacagctccctttgaaactttcaagaaaatgggaaaacagacaggcatcatctactatgtgccagccggattcacttccaagatctgccccgtgaccgg<br>ctttgtcaaccagctgtaccctaaatatgagtcagtgagcaagtcccaggaatttttcagcaagttcgataagatctgttataatctggacaaggggtacttcg<br>agttttccttcgattacaagaacttcggcgacaaggccgctaaggggaaatggaccattgcctccttcggatctcgcctgatcaactttcgaaattccgataa<br>aaaccacaattgggacactagggaggtgtacccaaccaaggagctggaaaagctgctgaaagactactctatcgagtatggacatggcgaatgcatc<br>aaggcagccatctgtggcgagagtgataagaaatttttcgccaagctgacctcagtgctgaatacaatcctgcagatgcggaactcaaagaccgggaca<br>gaactggactatctgattagccccgtggctgatgtcaacggaaacttcttcgacagcagacaggcacccaaaaatatgcctcaggatgcagacgccaac<br>ggggcctaccacatcgggctgaagggactgatgctgctgggccggatcaagaacaatcaggaggggaagaagctgaacctggtcattaagaacgag<br>gaatacttcgagtttgtccagaatagaaataacaaaaggccggcggccacgaaaaaggccggccaggcaaaaaagaaaaaggGATCCGGA<br>ACCCGGGCTGACGCATTGGACGATTTTGATCTGGATATGCTGGGAAGTGACGCCCTCGATGATTTTGAC<br>CTTGACATGCTTGGTTCGGATGCCCTTGATGACTTTGACCTCGACATGCTCGGCAGTGACGCCCTTGAT<br>GATTTCGACCTGGACATGCTGATTAACTCTAGAAGTTCCGGATCTCCGAAAAAGAAACGCAAAGTTGGTA<br>GCCAGTACCTGCCCGACACCGACGACCGGCACCGGATCGAGGAAAAGCGGAAGCGGACCTACGAGAC<br>ATTCAAGAGCATCATGAAGAAGTCCCCCTTCAGCGGCCCCACCGACCCTAGACCTCCACCTAGAAGAAT<br>CGCCGTGCCCAGCAGATCCAGCGCCAGCGTGCCAAAACCTGCCCCCCAGCCTTACCCCTTCACCAGCA<br>GCCTGAGCACCATCAACTACGACGAGTTCCCTACCATGGTGTTCCCCAGCGGCCAGATCTCTCAGGCCT<br>CTGCTCTGGCTCCAGCCCCTCCTCAGGTGCTGCCTCAGGCTCCTGCTCCTGCACCAGCTCCAGCCATG<br>GTGTCTGCACTGGCTCAGGCACCAGCACCCGTGCCTGTGCTGGCTCCTGGACCTCCACAGGCTGTGGC<br>TCCACCAGCCCCTAAACCTACACAGGCCGGCGAGGGCACACTGTCTGAAGCTCTGCTGCAGCTGCAGT<br>TCGACGACGAGGATCTGGGAGCCCTGCTGGGAAACAGCACCGATCCTGCCGTGTTCACCGACCTGGCC<br>AGCGTGGACAACAGCGAGTTCCAGCAGCTGCTGAACCAGGGCATCCCTGTGGCCCCTCACACCACCGA<br>GCCCATGCTGATGGAATACCCCGAGGCCATCACCCGGCTCGTGACAGGCGCTCAGAGGCCTCCTGATC<br>CAGCTCCTGCCCCTCTGGGAGCACCAGGCCTGCCTAATGGACTGCTGTCTGGCGACGAGGACTTCAGC<br>TCTATCGCCGATATGGATTTCTCAGCCTTGCTGGGCTCTGGCAGCGGCAGCCGGGATTCCAGGGAAGG<br>GATGTTTTTGCCGAAGCCTGAGGCCGGCTCCGCTATTAGTGACGTGTTTGAGGGCCGCGAGGTGTGCC<br>AGCCAAAACGAATCCGGCCATTTCATCCTCCAGGAAGTCCATGGGCCAACCGCCCACTCCCCGCCAGC<br>CTCGCACCAACACCAACCGGTCCAGTACATGAGCCAGTCGGGTCACTGACCCCGGCACCAGTCCCTCA<br>GCCACTGGATCCAGCGCCCGCAGTGACTCCCGAGGCCAGTCACCTGTTGGAGGATCCCGATGAAGAGA<br>CGAGCCAGGCTGTCAAAGCCCTTCGGGAGATGGCCGATACTGTGATTCCCCAGAAGGAAGAGGCTGCA<br>ATCTGTGGCCAAATGGACCTTTCCCATCCGCCCCCAAGGGGCCATCTGGATGAGCTGACAACCACACTT<br>GAGTCCATGACCGAGGATCTGAACCTGGACTCACCCCTGACCCCGGAATTGAACGAGATTCTGGATACC<br>TTCCTGAACGACGAGTGCCTCTTGCATGCCATGCATATCAGCACAGGACTGTCCATCTTCGACACATCTC<br>TGTTTTCCGGATACCCATACGATGTTCCAGATTACGCTTATCCCTACGACGTGCCTGATTATGCATACCC<br>ATATGATGTCCCCGACTATGCCTAA
**SspC half2-split v2**	ATGGTGAAAGTAATTGGGCGCCGGTCCCTGGGAGTCCAACGAATCTTTGACATTGGTCTGCGTCAAGAT<br>CATAATTTTCTTCTTGCTAATGGAGCTATCGCAGCAAACTGCTTTAATgctaacaagttcaacgacgagatcaatctgctg<br>ctgaaggaaaaagcaaacgatgtgcacatcctgagcattgaccgaggagagcggcatctggcctactataccctggtggatggcaaagggaatatcat<br>taagcaggatacattcaacatcattggcaatgaccggatgaaaaccaactaccacgataaactggctgcaatcgagaaggatagagactcagctagg<br>aaggactggaagaaaatcaacaacattaaggagatgaaggaaggctatctgagccaggtggtccatgagattgcaaagctggtcatcgaatacaatg<br>ccattgtggtgttcgaggatctgaacttcggctttaagagggggcgctttaaggtggaaaaacaggtctatcagaagctggagaaaatgctgatcgaaaa<br>gctgaattacctggtgtttaaagataacgagttcgacaagaccggaggcgtcctgagagcctaccagctgacagctccctttgaaactttcaagaaaatgg<br>gaaaacagacaggcatcatctactatgtgccagccggattcacttccaagatctgccccgtgaccggctttgtcaaccagctgtaccctaaatatgagtca<br>gtgagcaagtcccaggaatttttcagcaagttcgataagatctgttataatctggacaaggggtacttcgagttttccttcgattacaagaacttcggcgacaa<br>ggccgctaaggggaaatggaccattgcctccttcggatctcgcctgatcaactttcgaaattccgataaaaaccacaattgggacactagggaggtgtac<br>ccaaccaaggagctggaaaagctgctgaaagactactctatcgagtatggacatggcgaatgcatcaaggcagccatctgtggcgagagtgataaga<br>aatttttcgccaagctgacctcagtgctgaatacaatcctgcagatgcggaactcaaagaccgggacagaactggactatctgattagccccgtggctgat<br>gtcaacggaaacttcttcgacagcagacaggcacccaaaaatatgcctcaggatgcagacgccaacggggcctaccacatcgggctgaagggactg<br>atgctgctgggccggatcaagaacaatcaggaggggaagaagctgaacctggtcattaagaacgaggaatacttcgagtttgtccagaatagaaataa<br>caaaaggccggcggccacgaaaaaggccggccaggcaaaaaagaaaaaggGATCCGGAACCCGGGCTGACGCATTGGAC<br>GATTTTGATCTGGATATGCTGGGAAGTGACGCCCTCGATGATTTTGACCTTGACATGCTTGGTTCGGATG<br>CCCTTGATGACTTTGACCTCGACATGCTCGGCAGTGACGCCCTTGATGATTTCGACCTGGACATGCTGA<br>TTAACTCTAGAAGTTCCGGATCTCCGAAAAAGAAACGCAAAGTTGGTAGCCAGTACCTGCCCGACACCG<br>ACGACCGGCACCGGATCGAGGAAAAGCGGAAGCGGACCTACGAGACATTCAAGAGCATCATGAAGAAG<br>TCCCCCTTCAGCGGCCCCACCGACCCTAGACCTCCACCTAGAAGAATCGCCGTGCCCAGCAGATCCAG<br>CGCCAGCGTGCCAAAACCTGCCCCCCAGCCTTACCCCTTCACCAGCAGCCTGAGCACCATCAACTACG<br>ACGAGTTCCCTACCATGGTGTTCCCCAGCGGCCAGATCTCTCAGGCCTCTGCTCTGGCTCCAGCCCCTC<br>CTCAGGTGCTGCCTCAGGCTCCTGCTCCTGCACCAGCTCCAGCCATGGTGTCTGCACTGGCTCAGGCA<br>CCAGCACCCGTGCCTGTGCTGGCTCCTGGACCTCCACAGGCTGTGGCTCCACCAGCCCCTAAACCTAC<br>ACAGGCCGGCGAGGGCACACTGTCTGAAGCTCTGCTGCAGCTGCAGTTCGACGACGAGGATCTGGGA<br>GCCCTGCTGGGAAACAGCACCGATCCTGCCGTGTTCACCGACCTGGCCAGCGTGGACAACAGCGAGTT<br>CCAGCAGCTGCTGAACCAGGGCATCCCTGTGGCCCCTCACACCACCGAGCCCATGCTGATGGAATACC<br>CCGAGGCCATCACCCGGCTCGTGACAGGCGCTCAGAGGCCTCCTGATCCAGCTCCTGCCCCTCTGGGA<br>GCACCAGGCCTGCCTAATGGACTGCTGTCTGGCGACGAGGACTTCAGCTCTATCGCCGATATGGATTTC<br>TCAGCCTTGCTGGGCTCTGGCAGCGGCAGCCGGGATTCCAGGGAAGGGATGTTTTTGCCGAAGCCTGA<br>GGCCGGCTCCGCTATTAGTGACGTGTTTGAGGGCCGCGAGGTGTGCCAGCCAAAACGAATCCGGCCAT<br>TTCATCCTCCAGGAAGTCCATGGGCCAACCGCCCACTCCCCGCCAGCCTCGCACCAACACCAACCGGT<br>CCAGTACATGAGCCAGTCGGGTCACTGACCCCGGCACCAGTCCCTCAGCCACTGGATCCAGCGCCCGC<br>AGTGACTCCCGAGGCCAGTCACCTGTTGGAGGATCCCGATGAAGAGACGAGCCAGGCTGTCAAAGCCC<br>TTCGGGAGATGGCCGATACTGTGATTCCCCAGAAGGAAGAGGCTGCAATCTGTGGCCAAATGGACCTTT<br>CCCATCCGCCCCCAAGGGGCCATCTGGATGAGCTGACAACCACACTTGAGTCCATGACCGAGGATCTG

	AACCTGGACTCACCCCTGACCCCGGAATTGAACGAGATTCTGGATACCTTCCTGAACGACGAGTGCCTC<br>TTGCATGCCATGCATATCAGCACAGGACTGTCCATCTTCGACACATCTCTGTTTTCCGGATACCCATACG<br>ATGTTCCAGATTACGCTTATCCCTACGACGTGCCTGATTATGCATACCCATATGATGTCCCCGACTATGC<br>CTAA
**SspC half2-split v3**	ATGGTGAAAGTAATTGGGCGCCGGTCCCTGGGAGTCCAACGAATCTTTGACATTGGTCTGCGTCAAGAT<br>CATAATTTTCTTCTTGCTAATGGAGCTATCGCAGCAAACTGCTTTAATagcaagggcagaccaaacctgcatacactgt<br>actggaaggccctgttcgatgagaggaatctgcaggacgtggtctataaactgaacggagaggccgaactgttttaccggaagcagtctattcctaagaa<br>aatcactcacccagctaaggaggccatcgctaacaagaacaaggacaatcctaagaaagagagcgtgttcgaatacgatctgattaaggacaagcg<br>gttcaccgaagataagttctttttccattgtccaatcaccattaacttcaagtcaagcggcgctaacaagttcaacgacgagatcaatctgctgctgaaggaa<br>aaagcaaacgatgtgcacatcctgagcattgaccgaggagagcggcatctggcctactataccctggtggatggcaaagggaatatcattaagcagga<br>tacattcaacatcattggcaatgaccggatgaaaaccaactaccacgataaactggctgcaatcgagaaggatagagactcagctaggaaggactgg<br>aagaaaatcaacaacattaaggagatgaaggaaggctatctgagccaggtggtccatgagattgcaaagctggtcatcgaatacaatgccattgtggtg<br>ttcgaggatctgaacttcggctttaagagggggcgctttaaggtggaaaaacaggtctatcagaagctggagaaaatgctgatcgaaaagctgaattacc<br>tggtgtttaaagataacgagttcgacaagaccggaggcgtcctgagagcctaccagctgacagctccctttgaaactttcaagaaaatgggaaaacaga<br>caggcatcatctactatgtgccagccggattcacttccaagatctgccccgtgaccggctttgtcaaccagctgtaccctaaatatgagtcagtgagcaagt<br>cccaggaatttttcagcaagttcgataagatctgttataatctggacaaggggtacttcgagttttccttcgattacaagaacttcggcgacaaggccgctaa<br>ggggaaatggaccattgcctccttcggatctcgcctgatcaactttcgaaattccgataaaaaccacaattgggacactagggaggtgtacccaaccaag<br>gagctggaaaagctgctgaaagactactctatcgagtatggacatggcgaatgcatcaaggcagccatctgtggcgagagtgataagaaatttttcgcca<br>agctgacctcagtgctgaatacaatcctgcagatgcggaactcaaagaccgggacagaactggactatctgattagccccgtggctgatgtcaacggaa<br>acttcttcgacagcagacaggcacccaaaaatatgcctcaggatgcagacgccaacggggcctaccacatcgggctgaagggactgatgctgctggg<br>ccggatcaagaacaatcaggaggggaagaagctgaacctggtcattaagaacgaggaatacttcgagtttgtccagaatagaaataacaaaaggcc<br>ggcggccacgaaaaaggccggccaggcaaaaaagaaaaaggGATCCGGAACCCGGGCTGACGCATTGGACGATTTTGA<br>TCTGGATATGCTGGGAAGTGACGCCCTCGATGATTTTGACCTTGACATGCTTGGTTCGGATGCCCTTGAT<br>GACTTTGACCTCGACATGCTCGGCAGTGACGCCCTTGATGATTTCGACCTGGACATGCTGATTAACTCTA<br>GAAGTTCCGGATCTCCGAAAAAGAAACGCAAAGTTGGTAGCCAGTACCTGCCCGACACCGACGACCGG<br>CACCGGATCGAGGAAAAGCGGAAGCGGACCTACGAGACATTCAAGAGCATCATGAAGAAGTCCCCCTT<br>CAGCGGCCCCACCGACCCTAGACCTCCACCTAGAAGAATCGCCGTGCCCAGCAGATCCAGCGCCAGCG<br>TGCCAAAACCTGCCCCCCAGCCTTACCCCTTCACCAGCAGCCTGAGCACCATCAACTACGACGAGTTCC<br>CTACCATGGTGTTCCCCAGCGGCCAGATCTCTCAGGCCTCTGCTCTGGCTCCAGCCCCTCCTCAGGTG<br>CTGCCTCAGGCTCCTGCTCCTGCACCAGCTCCAGCCATGGTGTCTGCACTGGCTCAGGCACCAGCACC<br>CGTGCCTGTGCTGGCTCCTGGACCTCCACAGGCTGTGGCTCCACCAGCCCCTAAACCTACACAGGCCG<br>GCGAGGGCACACTGTCTGAAGCTCTGCTGCAGCTGCAGTTCGACGACGAGGATCTGGGAGCCCTGCTG<br>GGAAACAGCACCGATCCTGCCGTGTTCACCGACCTGGCCAGCGTGGACAACAGCGAGTTCCAGCAGCT<br>GCTGAACCAGGGCATCCCTGTGGCCCCTCACACCACCGAGCCCATGCTGATGGAATACCCCGAGGCCA<br>TCACCCGGCTCGTGACAGGCGCTCAGAGGCCTCCTGATCCAGCTCCTGCCCCTCTGGGAGCACCAGGC<br>CTGCCTAATGGACTGCTGTCTGGCGACGAGGACTTCAGCTCTATCGCCGATATGGATTTCTCAGCCTTG<br>CTGGGCTCTGGCAGCGGCAGCCGGGATTCCAGGGAAGGGATGTTTTTGCCGAAGCCTGAGGCCGGCT<br>CCGCTATTAGTGACGTGTTTGAGGGCCGCGAGGTGTGCCAGCCAAAACGAATCCGGCCATTTCATCCTC<br>CAGGAAGTCCATGGGCCAACCGCCCACTCCCCGCCAGCCTCGCACCAACACCAACCGGTCCAGTACAT<br>GAGCCAGTCGGGTCACTGACCCCGGCACCAGTCCCTCAGCCACTGGATCCAGCGCCCGCAGTGACTCC<br>CGAGGCCAGTCACCTGTTGGAGGATCCCGATGAAGAGACGAGCCAGGCTGTCAAAGCCCTTCGGGAGA<br>TGGCCGATACTGTGATTCCCCAGAAGGAAGAGGCTGCAATCTGTGGCCAAATGGACCTTTCCCATCCGC<br>CCCCAAGGGGCCATCTGGATGAGCTGACAACCACACTTGAGTCCATGACCGAGGATCTGAACCTGGAC<br>TCACCCCTGACCCCGGAATTGAACGAGATTCTGGATACCTTCCTGAACGACGAGTGCCTCTTGCATGCC<br>ATGCATATCAGCACAGGACTGTCCATCTTCGACACATCTCTGTTTTCCGGATACCCATACGATGTTCCAG<br>ATTACGCTTATCCCTACGACGTGCCTGATTATGCATACCCATATGATGTCCCCGACTATGCCTAA

**SspC half2-split v4**	ATGGTGAAAGTAATTGGGCGCCGGTCCCTGGGAGTCCAACGAATCTTTGACATTGGTCTGCGTCAAGAT<br>CATAATTTTCTTCTTGCTAATGGAGCTATCGCAGCAAACTGCTTTAATcacatcctgagcattgcgcgaggagagcggc<br>atctggcctactataccctggtggatggcaaagggaatatcattaagcaggatacattcaacatcattggcaatgaccggatgaaaaccaactaccacga<br>taaactggctgcaatcgagaaggatagagactcagctaggaaggactggaagaaaatcaacaacattaaggagatgaaggaaggctatctgagcca<br>ggtggtccatgagattgcaaagctggtcatcgaatacaatgccattgtggtgttcgaggatctgaacttcggctttaagagggggcgctttaaggtggaaaa<br>acaggtctatcagaagctggagaaaatgctgatcgaaaagctgaattacctggtgtttaaagataacgagttcgacaagaccggaggcgtcctgagagc<br>ctaccagctgacagctccctttgaaactttcaagaaaatgggaaaacagacaggcatcatctactatgtgccagccggattcacttccaagatctgccccg<br>tgaccggctttgtcaaccagctgtaccctaaatatgagtcagtgagcaagtcccaggaatttttcagcaagttcgataagatctgttataatctggacaaggg<br>gtacttcgagttttccttcgattacaagaacttcggcgacaaggccgctaaggggaaatggaccattgcctccttcggatctcgcctgatcaactttcgaaatt<br>ccgataaaaaccacaattgggacactagggaggtgtacccaaccaaggagctggaaaagctgctgaaagactactctatcgagtatggacatggcga<br>atgcatcaaggcagccatctgtggcgagagtgataagaaatttttcgccaagctgacctcagtgctgaatacaatcctgcagatgcggaactcaaagacc<br>gggacagaactggactatctgattagccccgtggctgatgtcaacggaaacttcttcgacagcagacaggcacccaaaaatatgcctcaggatgcaga<br>cgccaacggggcctaccacatcgggctgaagggactgatgctgctgggccggatcaagaacaatcaggaggggaagaagctgaacctggtcattaa<br>gaacgaggaatacttcgagtttgtccagaatagaaataacaaaaggccggcggccacgaaaaaggccggccaggcaaaaaagaaaaaggGAT<br>CCGGAACCCGGGCTGACGCATTGGACGATTTTGATCTGGATATGCTGGGAAGTGACGCCCTCGATGATT<br>TTGACCTTGACATGCTTGGTTCGGATGCCCTTGATGACTTTGACCTCGACATGCTCGGCAGTGACGCCC<br>TTGATGATTTCGACCTGGACATGCTGATTAACTCTAGAAGTTCCGGATCTCCGAAAAAGAAACGCAAAGT<br>TGGTAGCCAGTACCTGCCCGACACCGACGACCGGCACCGGATCGAGGAAAAGCGGAAGCGGACCTAC<br>GAGACATTCAAGAGCATCATGAAGAAGTCCCCCTTCAGCGGCCCCACCGACCCTAGACCTCCACCTAGA<br>AGAATCGCCGTGCCCAGCAGATCCAGCGCCAGCGTGCCAAAACCTGCCCCCCAGCCTTACCCCTTCAC<br>CAGCAGCCTGAGCACCATCAACTACGACGAGTTCCCTACCATGGTGTTCCCCAGCGGCCAGATCTCTCA<br>GGCCTCTGCTCTGGCTCCAGCCCCTCCTCAGGTGCTGCCTCAGGCTCCTGCTCCTGCACCAGCTCCAG<br>CCATGGTGTCTGCACTGGCTCAGGCACCAGCACCCGTGCCTGTGCTGGCTCCTGGACCTCCACAGGCT<br>GTGGCTCCACCAGCCCCTAAACCTACACAGGCCGGCGAGGGCACACTGTCTGAAGCTCTGCTGCAGCT<br>GCAGTTCGACGACGAGGATCTGGGAGCCCTGCTGGGAAACAGCACCGATCCTGCCGTGTTCACCGACC<br>TGGCCAGCGTGGACAACAGCGAGTTCCAGCAGCTGCTGAACCAGGGCATCCCTGTGGCCCCTCACACC<br>ACCGAGCCCATGCTGATGGAATACCCCGAGGCCATCACCCGGCTCGTGACAGGCGCTCAGAGGCCTCC<br>TGATCCAGCTCCTGCCCCTCTGGGAGCACCAGGCCTGCCTAATGGACTGCTGTCTGGCGACGAGGACT<br>TCAGCTCTATCGCCGATATGGATTTCTCAGCCTTGCTGGGCTCTGGCAGCGGCAGCCGGGATTCCAGG<br>GAAGGGATGTTTTTGCCGAAGCCTGAGGCCGGCTCCGCTATTAGTGACGTGTTTGAGGGCCGCGAGGT<br>GTGCCAGCCAAAACGAATCCGGCCATTTCATCCTCCAGGAAGTCCATGGGCCAACCGCCCACTCCCCG<br>CCAGCCTCGCACCAACACCAACCGGTCCAGTACATGAGCCAGTCGGGTCACTGACCCCGGCACCAGTC<br>CCTCAGCCACTGGATCCAGCGCCCGCAGTGACTCCCGAGGCCAGTCACCTGTTGGAGGATCCCGATGA<br>AGAGACGAGCCAGGCTGTCAAAGCCCTTCGGGAGATGGCCGATACTGTGATTCCCCAGAAGGAAGAGG<br>CTGCAATCTGTGGCCAAATGGACCTTTCCCATCCGCCCCCAAGGGGCCATCTGGATGAGCTGACAACCA<br>CACTTGAGTCCATGACCGAGGATCTGAACCTGGACTCACCCCTGACCCCGGAATTGAACGAGATTCTGG<br>ATACCTTCCTGAACGACGAGTGCCTCTTGCATGCCATGCATATCAGCACAGGACTGTCCATCTTCGACAC<br>ATCTCTGTTTTCCGGATACCCATACGATGTTCCAGATTACGCTTATCCCTACGACGTGCCTGATTATGCAT<br>ACCCATATGATGTCCCCGACTATGCCTAA
